# Receptor-independent regulation of Gα13 by alpha-1-antitrypsin C-terminal peptides

**DOI:** 10.1016/j.jbc.2024.108136

**Published:** 2024-12-25

**Authors:** Yonghak Park, Shigeyuki Matsumoto, Kosuke Ogata, Biao Ma, Ryo Kanada, Yuta Isaka, Norihito Arichi, Xiaowen Liang, Ritsuko Maki, Tohru Kozasa, Yasushi Okuno, Hiroaki Ohno, Yasushi Ishihama, Fumiko Toyoshima

**Affiliations:** 1 Department of Biosystems Science, Institute for Life and Medical Sciences, Kyoto University, Kyoto, Japan; 2 Department of Mammalian and Regulatory Networks, Graduate School of Biostudies, Kyoto University, Kyoto, Japan; 3 Department of Biomedical Data Intelligence, Graduate School of Medicine, Kyoto University, Kyoto, Japan; 4 Department of Molecular Systems BioAnalysis, Graduate School of Pharmaceutical Sciences, Kyoto University, Kyoto, Japan; 5 HPC- and AI-driven Drug Development Platform Division, RIKEN Center for Computational Science, Kobe, Hyogo, Japan; 6 Department of Bioorganic Medicinal Chemistry, Graduate School of Pharmaceutical Sciences, Kyoto University, Kyoto, Japan; 7 Department of Biochemistry, Yokohama University of Pharmacy, Yokohama, Japan; 8 Department of Homeostatic Medicine, Medical Research Laboratory, Institute of Integrated Research, Institute of Science Tokyo, Tokyo, Japan

**Keywords:** serpin, peptides, g protein, structural model, cell signaling, alpha-1-antitrypsin

## Abstract

Alpha-1-antitrypsin (AAT), a circulating serine protease inhibitor, is an acute inflammatory response protein with anti-inflammatory functions. The C-terminal peptides of AAT are found in various tissues and have been proposed as putative bioactive peptides with multiple functions, but its mechanism of action remains unclear. We previously reported that a mouse AAT C-terminal peptide of 35 amino acids (mAAT-C_1-35_) penetrates plasma membrane and associates guanine nucleotide-binding protein subunit alpha 13 (Gα13). Here, we show that mAAT-C_1-35_ binds directly to the guanosine diphosphate (GDP)-bound form of Gα13 through the N-terminal region (mAAT-C_1-17_), thereby facilitating the interaction of Gα13・GDP with its effector proteins. The minimal sequence (mAAT-C_3-16_) and essential amino acid residue (Phe11) of mAAT-C_1-17_ were identified as being necessary for this effect. A molecular dynamics simulation for the Gα13・GDP-mAAT-C_1-17_ complex model showed that binding of mAAT-C_1-17_ to the region surrounded by switch regions of Gα13 stabilizes the flexible switch II and III regions, thereby maintaining their active conformation. In addition, mAAT-C_1-35_ activates the Gα13 signaling pathway in cells where Phe11 is required. Our study reveals the structure-based mechanism of action of AAT-C peptides in the regulation of Gα13 and demonstrates that AAT-C peptides represent a biological peptide capable of activating G protein signals in a manner that is independent of G-protein-coupled receptors.

Alpha-1-antitrypsin (AAT), which belongs to the serine protease inhibitor (serpin) family A (SERPINA), is an acute-phase protein produced primarily in hepatocytes whose concentration in bloodstream increases during periods of inflammation (1, 2). AAT is well known as an inhibitor of human neutrophil elastase (HNE), which degrades extracellular matrix proteins including elastin, collagen, and proteoglycans (3, 4), thus preventing elastase-induced tissue destruction upon inflammation (5, 6). In the inhibition reaction, HNE binds to and cleaves human AAT at the site of Met382-Ser383 in the reactive center loop, generating AAT C-terminal peptides of 36 amino acids. This results in a conformational change of the complex, leading to the irreversible inactivation of HNE (7). In addition to HNE, a variety of non-target proteases, including matrix metalloproteases, cleave the carboxyl terminus of AAT, producing AAT C-terminal peptides of multiple lengths (8). Some of these peptides have been detected in human tissues and fluids and proposed as inflammatory disease biomarkers (9, 10, 11, 12, 13, 14). Despite its potential pathological activity, recent studies have identified many biological functions associated with AAT C-terminal peptides. These include the suppression of natural killer cells (15), the protection of extracellular matrix (16), and the inhibition of HIV entry (17). In a previous study, we showed that the 35-amino-acid length of the C-terminal peptide of the mouse AAT 1 to 2 protein (Serpin A1b), which we termed the tight junction-inducing peptide (JIP), can penetrate the plasma membrane and strengthen the tight junction *via* the guanine nucleotide-binding protein subunit alpha 13 (Gα13) (18). However, the mechanism of Gα13 regulation by AAT C-terminal peptides remains to be elucidated.

Gα13 is an alpha subunit of the Gα12/13 subfamily of the heterotrimeric G proteins. To date, more than 20 G protein-coupled receptors (GPCRs), including sphingosine-1-phosphate receptor2 (S1PR2) (19), thrombin protease-activated receptor-1 (PAR1) (20) and lysophosphatidic acid receptor 4 (LPA4) (21), have been identified as coupling to Gα13 (22, 23). In the canonical GPCR-mediated G protein signaling pathway, GPCRs are associated with inactive guanosine diphosphate (GDP)-bound Gα in a complex with Gβγ, which couples GPCR and Gα・GDP on the plasma membrane and also acts as a guanine nucleotide dissociation inhibitor (GDI) to inhibit the spontaneous release of GDP from Gα. Upon ligand activation, GPCRs function as guanine nucleotide exchange factors (GEFs), facilitating the exchange of GDP for guanosine triphosphate (GTP) on the Gα subunit. The binding of GTP induces a conformational change in three flexible switch regions on Gα, which in turn results in the dissociation of the Gαβγ complex into Gα・GTP and Gβγ subunits. Subsequently, both Gα・GTP and Gβγ regulate the downstream signals (24, 25, 26, 27). The Gα13・GTP has been demonstrated to directly bind and activate Rho guanine nucleotide exchange factors (RhoGEFs), including p115-RhoGEF and leukemia-associated RhoGEF (LARG), thereby leading to the activation of the RhoA signaling pathway (28, 29, 30, 31, 32). Upon signal termination, Gα・GTP is hydrolyzed to Gα・GDP by the intrinsic GTPase activity of Gα, which is accelerated by GTPase-activating protein (GAP) (33). The N-terminal regions including the regulators of the G protein signaling (RGS) homology (RH) domain of p115-RhoGEF and LARG act as a GAP for Gα13, allowing reversible regulation between Gα13 and p115-RhoGEF or LARG (34, 35, 36).

In addition to the well-established GPCR-mediated activation of G proteins, there is increasing body of evidence to suggest that GPCR-independent activation of G proteins also occurs (37). These include Gα-interacting Vesicle-associated (GIV), also known as GIRDers of actIN filament (Girdin), a first identified member of the guanine nucleotide exchange modulators (GEMs), which acts as a non-receptor cytosolic GEF for Gi (38) and as a GDI for Gs *via* the same evolutionarily conserved GEM motif (39). However, there is no evidence to date of GPCR-independent activation of Gα13. This study shows that AAT C-terminal peptides can activate Gα13 signaling independent of GPCRs.

## Results

### AAT C-terminal peptides bind and stabilize the association of G**α**13 with its effector proteins independently of GTP

**Figure 1:**
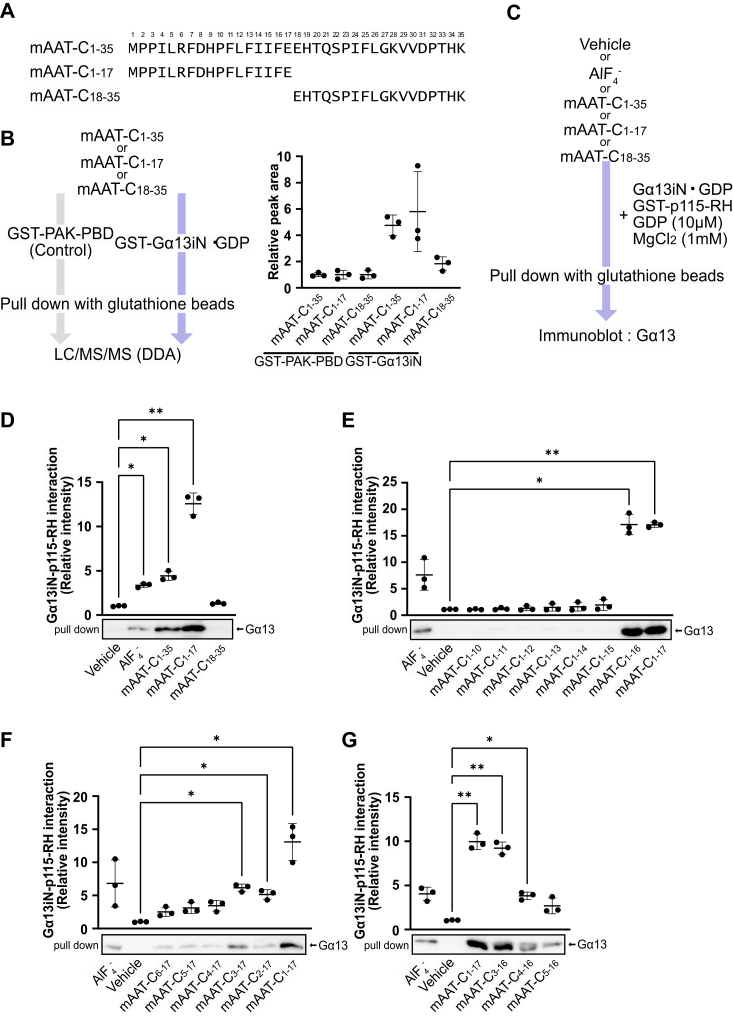
**AAT-C peptides bind and stabilize the association of Gα13・GDP with its effectors in the absence of GTP**. *A*, amino acid sequences of the mAAT-C peptides. *B*, AP-MS to analyze the binding of mAAT-C peptides and GST-Gα13iN protein. *C*, scheme of the GST pull-down assay to analyze the binding of Gα13・GDP and GST-p115-RH. *D*, GST pull-down assay for Gα13・GDP to GST-p115-RH with AlF_4_^-^ or mAAT-C peptides. The precipitates were immunoblotted with Gα13 antibodies. Quantitative values for the Gα13iN bands are shown in the upper graph (n = 3 independent samples). *E–G*, GST pull-down assay for Gα13・GDP and GST-p115-RH with a deletion series of mAAT-C peptides. Quantitative values for the Gα13iN bands are shown in the upper graphs (n = 3 independent samples). Data were compared using Dunnett's multiple comparison test (mean ± S.D.). ∗*p* < 0.05, ∗∗*p* < 0.01.

We have previously shown that the 35-amino acid length of the mouse AAT C-terminal peptide (mAAT-C_1-35_) derived from Serpin A1b penetrates the plasma membrane and associates with Gα13 in cells (18). Among six *Serpina1* paralogs in the mouse genome, in this study we used mAAT-C_1-35_ derived from the mouse AAT 1-1 protein (Serpin A1a) (Fig. 1*A*), the best studied Serpin A1 protein, which has an amino acid substitution at Val26 of Serpin A1b-mAAT-C_1-35_ to Leu. To gain insight into the mechanism by which mAAT-C_1-35_ regulates Gα13 function, we investigated whether mAAT-C_1-35_ directly binds to Gα13 *in vitro* using affinity purification mass spectrometry (AP-MS) (Fig. 1*B*). We purified a glutathione *S*-transferase (GST)-tagged recombinant Gα13iN protein, a chimeric Gα13 protein in which the αN helix of mouse Gα13 was replaced by that of rat Gαi to increase the protein solubility (40). The affinity purification of mAAT-C_1-35_ with the Gα13iN・GDP protein as bait resulted in a significantly higher peak area for mAAT-C_1-35_ compared to the control protein, GST-p21 binding domain of human p21 activated kinase 1 (PAK1-PBD), indicating a direct binding between mAAT-C_1-35_ and Gα13iN (Fig. 1*B*). Furthermore, it was observed that mAAT-C_1-17_, but not mAAT-C_18-35_ (the N- and C-terminal halves of mAAT-C_1-35_, respectively), binds to GST-Gα13iN (Fig. 1, *A* and *B*). These results demonstrate that mAAT-C_1-35_ directly binds to Gα13iN through its N-terminal half.

We next examined whether mAAT-C_1-17_ exerts an influence on the GTPase cycle of Gα13iN. To this end, the GTPase Glo assay (Promega), a commercially available assay kit for the measurement of GTPase, GAP, and GEF activities for G proteins (41, 42), was employed. Although the GTPase Glo assay is unlikely to be suitable for defining each step of the GTPase cycle of Gα13, it is useful for assessing the involvement of the molecules of interest in the overall GTPase cycle. In line with previous studies, the GTPase activity of Gα13iN, quantified by the consumption of GTP in the reaction buffer, was significantly increased in the presence of the N terminal region (1–246) of p115-RhoGEF including the RH domain (p115-RhoGEF-RH), which acts as a GAP for Gα13 (34) (Fig. S1*A*). In contrast, no effect of mAAT-C_1-17_ on the GTPase cycle of GST-Gα13iN was detected by using either GTPase/GAP reaction buffer (low Mg^2+^ plus EDTA) (Fig. S1*B*) or GEF reaction buffer (high Mg^2+^) (Fig. S1*C*). These results suggest that mAAT-C_1-17_ is unlikely to affect the GTPase cycle of Gα13iN.

Then, we investigated whether the binding of mAAT-C_1-17_ induces a conformational change in Gα13・GDP, leading to its association with its downstream target proteins in the absence of GTP. To test this hypothesis, the interaction between Gα13iN・GDP and its target protein p115-RhoGEF-RH was assessed by GST pull-down assay with or without mAAT-C peptides (Fig. 1*C*). In line with previous studies (40, 43, 44), Gα13iN forms a complex with the p115-RhoGEF-RH in the presence of GDP and aluminum fluoride (AlF_4_^-^), which serves as a transition state analogue of GTP hydrolysis (45) but not with GDP alone (Figs. 1*D* and S2*A*). Then, mAAT-C peptides were added to the reaction mixture instead of AlF_4_^-^. The results showed that mAAT-C_1-35_ and mAAT-C_1-17_, but not mAAT-C_18-35_, were capable of inducing the formation of a stable complex between Gα13iN and p115-RhoGEF-RH (Figs. 1*D* and S2*A*). LARG-RH, a target protein of Gα13, gave a similar result (Fig. S3, *A* and *B*). The GST pull-down assay using deletion mutants of mAAT-C_1-17_ identified mAAT-C_3-16_ as a minimum functional region for stabilizing Gα13iN-p115-RhoGEF-RH (Figs. 1, *E*–*G* and S2, *B*–*D*) and Gα13iN-LARG-RH complexes (Fig. S3, *C* and *D*). These results showed that mAAT-C_1-35_ interacts with Gα13・GDP through its N-terminus and induces the association of Gα13・GDP with its effector proteins.

### Phe11 residue of mAAT-C peptide is essential for stabilization of G**α**13・GDP and p115-RhoGEF-RH association

**Figure 2:**
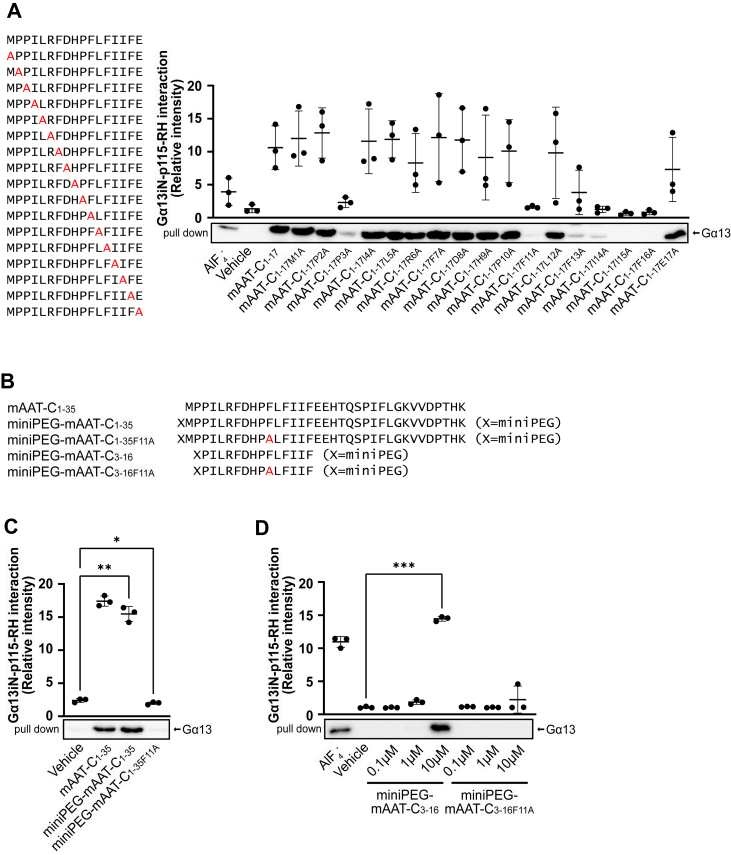
**Phe11 residue of mAAT-C peptides is essential for stabilization of Gα13・GDP-p115-RhoGEF-RH association**. *A*, GST pull-down assay for Gα13・GDP and GST-p115-RH with alanine-scan peptides of mAAT-C_1-17_. Quantitative values for the Gα13iN bands are shown in the graph (n = 3 independent samples). *B*, amino acid sequences of the miniPEG-mAAT-C peptides. *C* and *D*, GST pull-down assay for Gα13・GDP and GST-p115-RH with miniPEG-mAAT-C peptides. Quantitative values for the Gα13iN bands are shown in the graph (n = 3 independent samples). Data were compared using Dunnett's multiple comparison test (mean ± S.D.) (*A*, *C*, and *D*). ∗*p* < 0.05, ∗∗*p* < 0.01, ∗∗∗*p* < 0.001. miniPEG = H_2_N(CH_2_CH_2_O)_2_CH_2_CO.

To identify the amino acid residues in mAAT-C_1-17_ that are essential for Gα13 regulation, the alanine scanning of mAAT-C_1-17_ was subjected to the GST pull-down assay. Among the 17 amino acids, substitutions of Pro3, Phe11, Phe13, Ile14, Ile15 or Phe16 with Ala resulted in a reduction in the stabilizing activity of mAAT-C_1-17_ for Gα13 and p115-RhoGEF-RH association (Figs. 2*A* and S4*A*). Among them, Pro3, Phe11, and Phe13 exhibited conservation across five mouse Serpin A1 families (Serpin A1a-e) and human AAT. Since the substitution of Phe11 for Ala consistently reduced the stabilizing activity more than Pro3 or Phe13 for Ala (Fig. 2*A*), we focused our analysis on Phe11. Then, mutant forms of the full length (mAAT-C_1-35_) and minimum functional length (mAAT-C_3-16_) of mAAT-C peptides, in which Phe11 was substituted by Ala, were designed. The peptides were further modified through the addition of a mini polyethylene glycol (miniPEG) unit [H_2_N(CH_2_CH_2_O)_2_CH_2_CO-] at the N-terminus, thereby enhancing their water solubility (Fig. 2*B*). The GST pull-down assay showed that both miniPEG-mAAT-C_1-35_F11A and miniPEG-mAAT-C_3-16_F11A exhibited diminished activity in comparison to the control miniPEG-mAAT-C_1-35_ and miniPEG-mAAT-C_3-16_, respectively (Figs. 2, *C* and *D* and S4, *B* and *C*). These results indicate that Phe11 of mAAT-C peptides plays a pivotal role in stabilizing Gα13・GDP and p115-RhoGEF-RH association.

### Binding of mAAT-C_1-17_ to G**α**13iN・GDP stabilizes the active conformation of the switch regions and the p115RhoGEF-RH interface of G**α**13iN

**Figure 3:**
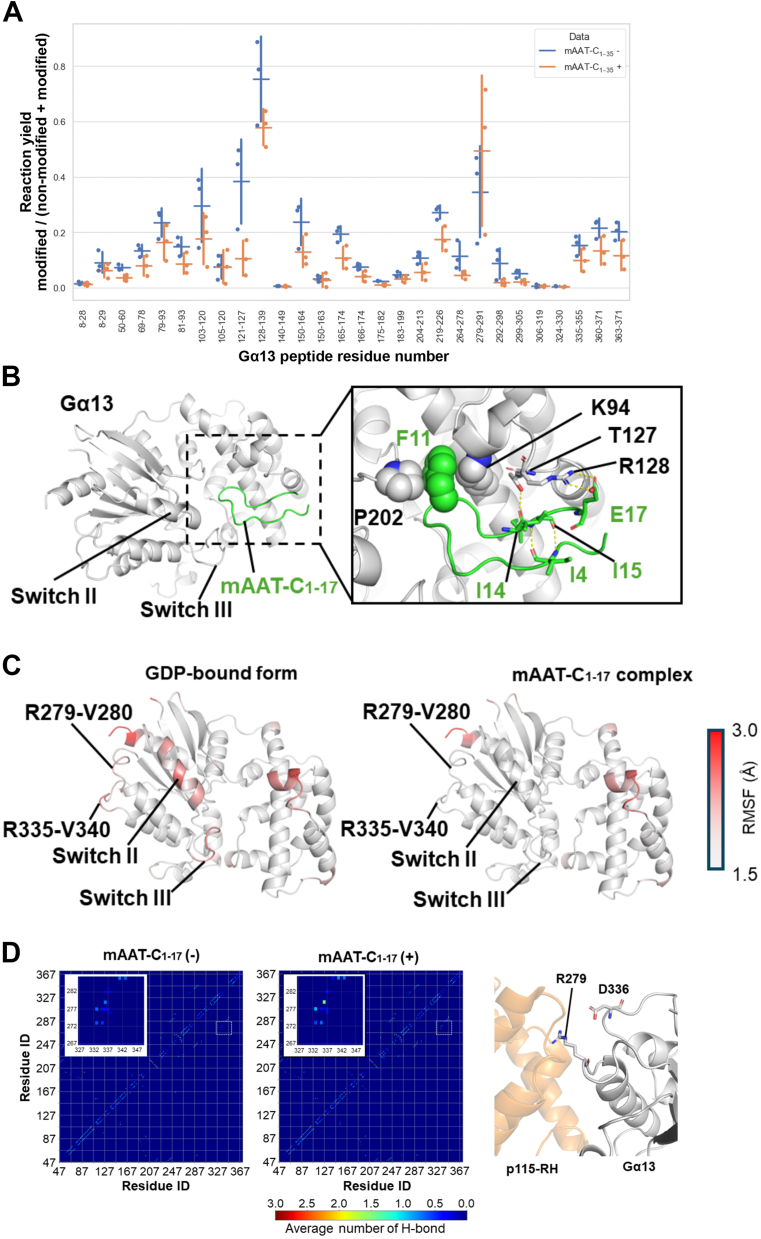
**MD simulations of the complex structure of mAAT-C**_**1-17**_**and Gα13・GDP.***A*, Diazirine-based protein footprinting for human Gα13 and Serpin A1b-mAAT-C_1-35_. The reaction yields of diazirine-based footprinting were calculated by the quantified peak areas of diazirine-modified peptides and corresponding non-modified peptides. The asterisks indicate peptides with *p*-value < 0.05 (Student’s *t* test) (mean ± S.D.). *B*, docking model of mAAT-C_1-17_ and Gα13・GDP. The *left panel* shows the overall view of the docking model, and the *right panel* shows close-up views of the regions indicated by the dashed rectangle in the overall view. In the close-up view, the residues forming hydrogen-bonding interactions and those forming hydrophobic interactions are indicated by *stick* and sphere models. The hydrogen bonding interactions are indicated by *yellow dashed lines*. *C*, mapping of RMSF values onto the tertiary structure of Gα13 (PDB entry 3AB3). The RMSF values are indicated by a gradual color change, from *grey* (1.5 Å) to *red* (3.0 Å). *D*, Hydrogen bonding interactions within Gα13. The number of hydrogen bond interactions is calculated for all residue pairs formed within Gα13. The averages across all MD trajectories in the absence (*left panel*) and presence (*middle panel*) of mAAT-C_1-17_ are shown as heatmaps with a color gradient from *blue* (*low*) to *red* (*high*). The *horizontal and vertical axes* represent the residue number. The inset shows enlarged views at the regions indicated by dashed rectangles in the overall heatmaps. The *right panel* shows a close-up view of the interface between Gα13 and p115-RH. Gα13 and the p115-RH are colored by *grey* and *orange*, respectively. Arg279 and Asp336 of Gα13, which form stable hydrogen bond interactions in the presence of mAAT-C_1-17_, are indicated by *stick models*.

To gain insight into the molecular mechanism underlying the association of Gα13・GDP and p115-RhoGEF-RH by mAAT-C_1-17_, molecular dynamics (MD) simulations were performed on the Gα13iN・GDP-mAAT-C_1-17_ complex. Firstly, diazirine-based protein foot printing was performed by using GST-human Gα13 and mAAT-C_1-35_ derived from Serpin A1b (not Serpin A1a) to identify accessible protein surfaces induced by the peptide binding (46) (Fig. 3*A*). GST-human Gα13 was incubated with or without mAAT-C_1-35_ peptides and was labelled using a diazirine reagent. After the labelling reaction, the protein was digested using trypsin, and the resultant tryptic peptides were analyzed by mass spectrometry to calculate the difference in foot printing labelling yield for each peptide. As a result, it was found that the reaction yield decreased in the presence of the mAAT-C_1-35_ peptide for 26 of the 28 quantified peptides from Gα13, indicating that the mAAT-C_1-35_ peptide restricts access to almost all of the surface of the Gα13 protein. Among these quantified peptides, 6 peptides with a *p*-value <0.05, corresponding to Gα13 residue numbers 50-60, 121-127, 165-174, 175-182, 219-226, and 299-305, were selected for subsequent docking simulation analysis with the program, HADDOCK2.4 (47). Since the foot printing experiments could identify both direct and indirect shielding effects for each amino acid residue from bulk solvent, all of these 6 peptides were used for ambiguous interaction restraints in the docking simulation.

The initial structures of mAAT-C_1-17_ were derived from Temperature-Replica Exchange Molecular Dynamics (T-REMD) simulation starting from the extended structure. The T-REMD simulation was performed to explore the conformational space in solution, and the stable conformations of mAAT-C_1-17_ were employed as the input structures (Fig. S5). The initial structure of Gα13iN・GDP was derived from the crystal structure of Gα13iN・GDP・AlF_4_, a transiently active form of Gα13iN, in complex with the p115RhoGEF RH domain (PDB entry: 3AB3) (48), with the aim of evaluating the structural stabilization of the functionally essential switch regions as an active form in the subsequent MD simulations. The docking simulation generates 400 models with the HADDOCK scores. The root-mean-square deviation (RMSD)-based clustering with a 5.0 Å cutoff was performed on the models, resulting in 314 models clustering into 17 clusters (Fig. S6*A*). The representative structures derived from the top six clusters, which covered approximately 75% of the clustered models, showed that mAAT-C_1-17_ bound to two surfaces; the surface containing the three switch regions (switch I, Leu198-His207; switch II, Val223-Ser239; switch III, Gln254-Asn263) (Surface 1, two of six models) or the opposite surface (Surface 2, four of six models) (Fig. S6*B*). To evaluate their predicted binding modes, we performed 50 ns MD simulations using the representative models as initial structures. During the simulation periods, mAAT-C_1-17_ bound to Surface 1 stably maintained the initial binding modes whereas those bound to Surface 2 readily deviated (Fig. S6*C*), indicating that Surface 1 may be the promising binding site for mAAT-C_1-17_. Of the two binding modes at Surface 1, the one with the best docking score was adopted and used for further analysis. It should be noted that the score of the adopted model was the best of all the docking models.

The best docking model showed that mAAT-C_1-17_ adopts a bent conformation around its central Phe11 and binds to Gα13iN in the region surrounded by the three switch regions (Fig. 3*B*). The bent conformation of mAAT-C_1-17_ was stabilized by intramolecular hydrogen bonds between Ile4 and Ile14 and between Ile4 and Ile15. The binding of mAAT-C_1-17_ to Gα13iN was stabilized by several intermolecular interactions, including hydrophobic interactions of Phe11 with Pro202 in the switch I and Lys94 in the αA2 helix of Gα13iN, a hydrogen bond of Ile14 with Thr127 of Gα13iN, and a salt bridge of Glu17 with Arg128 of Gα13iN. Superimposition of the docking model with the crystal structure of the Gα13iN・GDP・AlF_4_-p115-RhoGEF-RH complex showed that the binding site of mAAT-C_1-17_ was distinct from that of p115-RhoGEF-RH domain (amino acid (aa) 46–234), but overlapped with the regions that binds to the N-terminal extension of p115-RhoGEF-RH (aa 22–34) (Fig. S7*A*). The N-terminal extension of p115-RhoGEF-RH is not necessary but facilitates the binding of p115-RhoGEF-RH to Gα13 (36), suggesting that binding of mAAT-C_1-17_ to this region allosterically induces a conformational change of Gα13iN at the interface with p115-RhoGEF-RH to induce their binding. In support of this hypothesis, we found that the p115-RhoGEF-RH domain (42–252), the RGS box which lacks the N-terminal extension, binds to Gα13iN・GDP in the presence of mAAT-C_1-17_, but not with mAAT-C_1-17_F11 A (Fig. S7, *B* and *C*).

To interrogate the structural stabilization of the Gα13iN・GDP-mAAT-C_1-17_ complex, using the docking model as an initial structure, we performed 500 ns MD simulations with five different initial velocities. Root-mean-square deviation (RMSD) analysis of the mAAT-C_1-17_ demonstrated that the bound peptide remained associated with Gα13iN・GDP during the entire simulation period, indicating that the docking structure was stable (Fig. S8*A*). Analysis of the hydrogen bonding patterns within mAAT-C_1-17_ showed that those found in the initial structure were maintained throughout the simulation period (Fig. S8*B*), indicating that the bent conformation of mAAT-C_1-17_ was stable. To investigate the effect of mAAT-C_1-17_ binding on Gα13iN・GDP, we performed 500 ns MD simulations for Gα13iN・GDP in the absence of mAAT-C_1-17_ and compared the dynamic behavior with that of the complex. Root-mean-square fluctuation (RMSF) analysis demonstrated that the mAAT-C_1-17_ binding suppressed the flexibility of catalytically essential switch II and III regions of Gα13iN (Figs. 3*C* and S8*C*). The RMSD distributions compared to the initial structure, *i.e.*, the active conformation, showed no remarkable difference in the total structure of Gα13iN・GDP in the presence or absence of mAAT-C_1-17_ (Fig. S9, top left panel). However, the RMSD distributions of the switch regions, especially switch II, of the Gα13iN・GDP in the absence of mAAT-C_1-17_ were observed to deviate from those in the presence of mAAT-C_1-17_ (Fig. S9, top right and bottom panels). Given that the most notable structural distinction between the active and inactive forms of Gα13iN was identified in switch regions, which adopt varying conformations in the GDP-bound form and are stabilized in the active conformation (40, 48), these findings imply that the structure of the mAAT-C_1-17_-bound form of Gα13iN・GDP resembles its active conformation. Conversely, the conformational transition to the inactive form is thought to have occurred in the absence of mAAT-C_1-17_.

In addition to the switch regions, the flexibility of the Arg279-Val280 and Arg335-Val340 of Gα13iN located at the interface with p115-RhoGEF-RH (48) was also suppressed in the presence of mAAT-C_1-17_ (Figs. 3*C* and S8*C*). Intriguingly, the average number of hydrogen bond interactions between Arg279 and Asp336 of Gα13iN was formed more frequently in the presence of mAAT-C_1-17_ than in the absence of mAAT-C_1-17_ during the simulation (Fig. 3*D*, 1.44 ± 0.18), suggesting that this hydrogen bond suppresses the flexibility of Gα13iN at the interface with p115-RhoGEF-RH. These results taken together demonstrate that the binding of mAAT-C_1-17_ stabilize the active conformation of switch regions and the interface with p115-RhoGEF-RH of Gα13iN.

Notably, the binding sites of Gα13iN to mAAT-C_1-17_ did not overlap with those to the Gβ1/Gγ2 subunits (Fig. S7*D*). However, the conformation of Gα13iN at the interface with Gβ1 was different between mAAT-C_1-17_ bound (this study) and Gβ1/Gγ2 bound (PDB entry: 7T6B) forms of Gα13iN (Fig. S7*D*, gray and black). These results suggest that the binding of mAAT-C_1-17_ may affect on the interaction between Gα13iN and Gβ1/Gγ.

### mAAT-C_1-35_ activates the G**α**13-RhoA signaling pathway in cells

**Figure 4:**
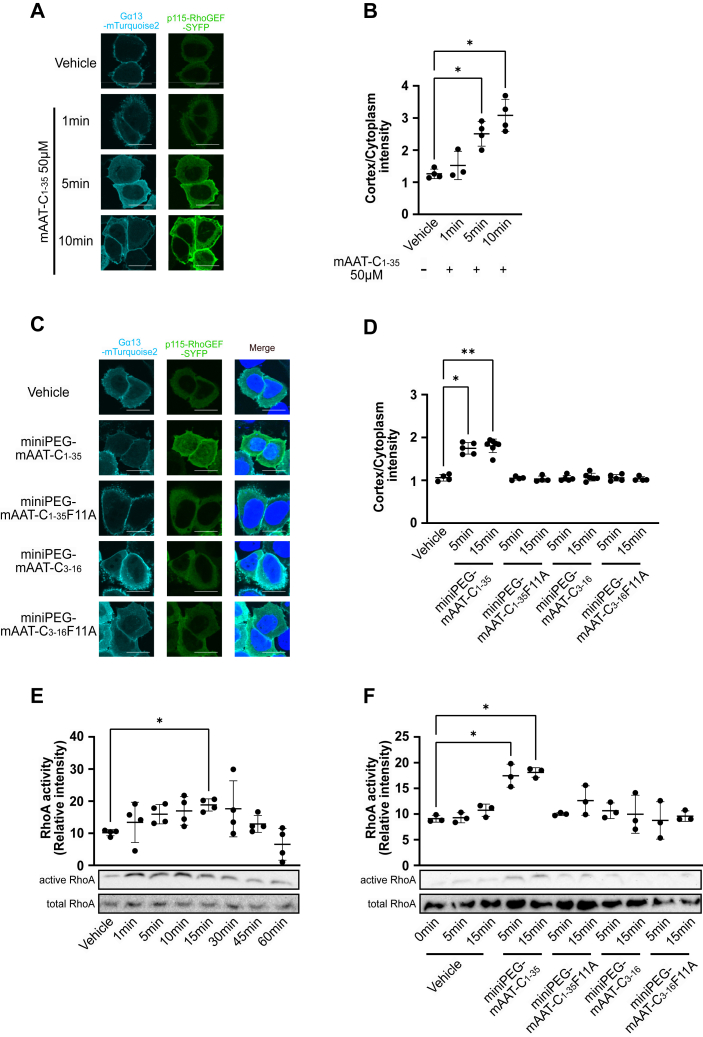
**mAAT-C**_**1-35**_**activates the Gα13-RhoA signaling pathway in cells.***A*, plasma membrane recruitment assay of p115-RhoGEF. HeLa cells transfected with Gα13-mTurquoise2 and SYFP1-p115-RhoGEF were treated with or without mAAT-C_1-35_ (50 μM) for the indicated time. *B*, the cortical intensity of SYFP1-p115-RhoGEF relative to the cytoplasmic intensity in (*A*) is shown. Each point represents the average of 11 to 14 cells/sample. *(n = 3 or 4 independent experiments)*. *C*, plasma membrane recruitment assay of p115-RhoGEF in cells treated with miniPEG-mAAT-C peptides (50 μM). *D*, cortical intensity of SYFP1-p115-RhoGEF relative to the cytoplasmic intensity in (*C*) is shown. Each point represents the average of 14 to 21 cells/sample. *(n > 4 independent experiments)*. *E* and *F*, GST-Rhotekin-RBD pull-down assay for RhoA activity in cells. HeLa cells treated with mAAT-C_1-35_ (50 μM) (*E*) or miniPEG-mAAT-C peptides (50 μM) (*F*) were lysed and the cell lysates were subjected to a GST-Rhotekin-RBD pull-down assay (active RhoA). Total cell lysates were used as loading control (total RhoA). Quantitative values for the active RhoA bands are shown in the graph *(n = 3 or 4 independent samples)*. Data were compared using Dunnett's multiple comparison test (mean ± S.D.) (*B*, *D*, *E*, and *F*). ∗*p* < 0.05, ∗∗*p* < 0.01. Scale bars, 20 μm (*A* and *C*).

The activation of Gα13 in cells results in the recruitment of p115-RhoGEF-RH to the plasma membrane (49). To evaluate the Gα13 activation capacity of mAAT-C_1-35_ in cellular contexts, HeLa cells were transfected with plasmids encoding YFP-tagged p115-RhoGEF-RH and mTurquoise2Δ9-tagged Gα13 (50) and treated with or without mAAT-C_1-35_. The recruitment of p115-RhoGEF-RH to the plasma membrane was significantly enhanced within 5 minutes of incubation with either mAAT-C_1-35_ (Fig. 4, *A* and *B*) or miniPEG-mAAT-C_1-35_ (Fig. 4, *C* and *D*). Treatment of the cells with miniPEG-mAAT-C_1-35_ without expression of mTurquoise2Δ9-tagged Gα13 also induced the recruitment of p115-RhoGEF-RH to the plasma membrane (Fig. S10, *A* and *B*), although the cortex/cytoplasmic intensity was lower than that with expression of mTurquoise2Δ9-tagged Gα13. These results suggest that both ectopic Gα13 and endogenous Gα13 can be activated by mAAT-C_1-35_ to recruit p115-RhoGEF-RH to the plasma membrane. In addition, both mAAT-C_1-35_ and miniPEG-mAAT-C_1-35_ were observed to induce RhoA activation in cells, which was monitored by active Rho pull-down assay utilizing Rho binding domain (RBD) of Rhotekin protein, within a five-minute treatment period (Figs. 4, *E* and *F* and S11, *A*, and *B*). In contrast, miniPEG-mAAT-C_1-35_F11 A was unable to induce the recruitment of p115-RhoGEF-RH to the plasma membrane (Figs. 4, *C* and *D* and S10, *A* and *B*) or to activate RhoA (Figs. 4*F* and S11*B*) in cells. These results demonstrate that mAAT-C_1-35_ activates Gα13-RhoA signaling within the cells, with the Phe11 of mAAT-C_1-35_ being critical for this function. It is noteworthy that mAAT-C_3-16_, a minimum functional sequence for Gα13 activation *in vitro*, was unable to induce the recruitment of p115-RhoGEF-RH to the plasma membrane (Fig. 4, *C* and *D*) or to activate RhoA (Figs. 4*F* and S11*B*) in cells. The results indicate that additional regions of mAAT-C_1-35_, in addition to mAAT-C_3-16_, are necessary for the *in vivo* activation of Gα13.

## Discussion

This study elucidates the mechanism by which AAT-C peptides modulate Gα13 signaling. The application of non-biased deletion/alanine scanning mutagenesis and MD simulations has revealed a previously unidentified mechanism of GPCR-independent structural regulation of Gα13 by AAT-C peptides. The mAAT-C peptides bind Gα13・GDP, thereby stabilizing its active conformation in the switch regions and the effector binding regions in the absence of GTP. This scenario is analogous to that observed with the GEM motif peptide of GIV/Girdin, which binds and activates Gαi・GDP independently of GPCRs (37, 38). However, the interface of the complex differs between the two: the GIV-GEM motif peptide interacts with the switch II region and the α3-helix of Gαi (51), whereas the mAAT-C_1-17_ insert into the hydrophobic pocket between the switch I region and the αA2-helix, and contact the helical insert region between the αB and the αB1-helices of Gα13. While MD simulations indicate that the binding of mAAT-C_1-17_ stabilizes the active conformation of the switch II region of Gα13, no effect is observed regarding the GTPase cycle of Gα13. This suggests that, in contrast to the GIV-GEM motif peptide, mAAT-C_1-17_ exerts a limited influence on the basal nucleotide exchange or GTP hydrolysis of Gα13. Instead, it facilitates the association of Gα13・GDP with the downstream effectors. The predicted binding site of mAAT-C_1-17_ was equivalent to the region that recognize an N-terminal extension of the RGS box in p115-RhoGEF-RH (48), suggesting that the N-terminal extension and mAAT-C_1-17_ have a similar role in stabilizing the scaffold for recognition of the downstream effectors. Since the RGS box of p115-RhoGEF-RH alone is sufficient to bind Gα13iN・GDP in the presence of AAT-C_1-17_ (Fig. S7, *B* and *C*), the interaction of the Gα13iN-mAAT-C_1-17_ complex with p115-RhoGEF-RH would be mediated by the RGS box, and the N-terminal extension of p115-RhoGEF-RH would not associate with Gα13iN in the ternary complex.

The binding site of mAAT-C_1-17_ in the docking model does not overlap with that of the Gβ1/Gγ2 subunits, but the switch II region, the interface between Gα13iN and Gβ1, adopted a different conformation in the presence of mAAT-C_1-17_ compared to that in the absence of mAAT-C_1-17_ (Fig. S7*D*). This observation predicts that Gβ1/Gγ2 subunits could not bind to the Gα13iN・GDP-mAAT-C_1-17_ complex. Biochemical and cell-based analyses in future studies are needed to evaluate this hypothesis. It would also be valuable to see whether mAAT-C_1-17_ could bind to a Gα13/Gβ/Gγ heterotrimer to induce Gβ/Gγ release, or whether Gα13 would need to be dissociated from Gβ/Gγ to allow mAAT-C_1-17_ access.

A limitation of the study is the short length of the MD simulation. The MD simulations in this study with a length of 500 ns, failed to capture the overall deviation from the active conformation in the absence of mAAT-C_1-17_ (Fig. S9). Large conformational changes, such as the structural transition between active and inactive forms, generally occur on timescales of millisecond or longer. The longer MD simulations using the dedicated machine, such as Anton (52), would be necessary to capture all the spontaneous conformational exchange events.

Despite the *in vivo* activation activity of Gα13, the functional minimum region (mAAT-C_3-16_) is unable to activate Gα13 in cells when introduced into the culture medium. This is presumably due to its lacking membrane permeability, which was observed in mAAT-C_1-35_ (18). It is possible that the sequences located outside the functional minimum region may be necessary for the penetration of the membrane. From this perspective, the multiple lengths of AAT-C peptides found in physiological and pathological conditions may exhibit differences in their membrane permeability, which may explain the functional differences observed.

Patients with AAT deficiency, caused by *SERPINA1* mutations, experience chronic lung and liver condition, such as emphysema and chronic obstructive pulmonary disease. It would be beneficial for future studies to investigate the potential involvement of mAAT-C peptides-Gα13 axis in the pathogenesis of AATD.

## Experimental procedures

### Peptide

Synthetic peptides were manufactured by PEPTIDE INSTITUTE INC (for Figs. 1, *D* and *G*, 2, *C* and *D*, 4, *A*–*F*, S1, *B* and *C*, S2, *A* and *D*, S3, *A*–*D*, S4, *B* and *C*, and S6, *A* and *B*) or SCRUM INC (for Figs. 1, *E* and *F*, 2*A*, and S4*A*). Each peptide was dissolved in 1% acetic acid (mAAT-C_1-35_, mAAT-C_18-35,_ mAAT-C_1-17_), 5% acetic acid (mAAT-C_1-10_, mAAT-C_1-11,_ mAAT-C_1-12_, mAAT-C_1-13,_ mAAT-C_1-14_, mAAT-C_1-15,_ mAAT-C_1-16_, mAAT-C_1-17,_ mAAT-C_3-16,_ mAAT-C_4-16_, mAAT-C_5-16,_ mAAT-C_1-17M1A_, mAAT-C_1-17P2A_, mAAT-C_1-17I4A_, mAAT-C_1-17L5A_, mAAT-C_1-17F7A_, mAAT-C_1-17D8A_, mAAT-C_1-17F11A_, mAAT-C_1-17L12A_, mAAT-C_1-17F13A_, mAAT-C_1-17L14A_, mAAT-C_1-17I15A_, mAAT-C_1-17F16A_, mAAT-C_1-17E17A_), DMSO (mAAT-C_6-17_, mAAT-C_5-17,_ mAAT-C_4-17_, mAAT-C_3-17,_ mAAT-C_2-17,_ mAAT-C_1-17,_ mAAT-C_1-17P3A,_ mAAT-C_1-17P10A_), 1% ammonia solution (mAAT-C_1-17R6A,_ mAAT-C_1-17H9A_), or distilled water (miniPEG-mAAT-C_1-35_, miniPEG-mAAT-C_1-35F11A,_ miniPEG-mAAT-C_3-16_, miniPEG-mAAT-C_3-16F11A)_.

### Expression constructs

The GST-Gα13iN chimera was prepared by the ligation of the N-terminal coding region of rat Gαi1 (amino acid residues 1–28) to Gα13 (amino acid residues 47–377) (40) and subcloning the product into pGEX-6P-1. The expression construct for GST-p115-RH or GST-LARG-RH was generated by subcloning the coding region of human p115RhoGEF (amino acid residues 1–252) or human LARG (amino acid residues 319–598) into pGEX-6P-1.

### Production of recombinant proteins

Plasmid DNAs were isolated using the QIAGEN Plasmid Mini Kit (QIAGEN, 12123) and introduced into BL21[DE3]. 500 ml LB medium (1% tryptone, 0.5% yeast extract, 1% NaCl supplemented with 100 μg/ml ampicillin) was inoculated with a colony of pGEX-6P-1-Gα13iN, pGEX-6P-1-p115-RH, pGEX-6P-1-p115-RH (42–252) or pGEX-6P-1-LARG-RH. The culture was incubated for 3 to 4 h at 37 °C with shaking, followed by the addition of 1 mM isopropyl-β-D-thiogalactopyranoside (IPTG) and the incubation at 18 °C for 18 h. Cell pellets were suspended in 20 ml of Tris buffer (50 mM Tris, 2 mM EGTA, 2 mM MgCl_2_, 2 mM dithiothreitol (DTT), 0.1 M phenylmethylsulfonyl fluoride (PMSF), pH 8.5), sonicated and centrifuged at 15,000 rpm for 20 min. The supernatants were incubated with glutathione-sepharose 4B beads (Cytvia, 17075601) for O/N at 4 °C. The recombinant proteins of GST-Gα13iN, GST-p115-RH, GST-p115-RH(42-252), and GST-LARG-RH were eluted from the beads with the elution buffer (50 mM Tris, 10 mM glutathione, 2 mM DTT, 1 mM PMSF, 2ug/ml aprotinin, pH9.5), followed by dialysis in the HEPES buffer (20 mM HEPES, 10 mM 2-mercaptoethanol (βME), 1 mM MgCl_2_, 100 mM NaCl, 10 μM GDP, pH 8.0). After dialysis, GDP was added to GST-Gα13iN at a final concentration of 10 μM. To generate untagged Gα13iN recombinant protein, GST-Gα13iN proteins were bound to the glutathione-sepharose 4B bead column, washed with Tris buffer, and incubated with PreScission Protease (Cytvia, 27084301) in PreScission Protease buffer (50 mM Tris, 100 mM NaCl, 1 mM EDTA, 1 mM DTT, pH 8.0) for O/N at 4 °C. The untagged Gα13iN was eluted and dialysed with the HEPES buffer using a cellulose dialysis tubular membrane (Viskase Companies Inc, 521713). After dialysis, GDP was added at a final concentration of 10 μM.

### Sample preparation for affinity purification mass spectrometry (AP-MS)

Affinity purification was performed using the MagneGST Protein Purification System (Promega, V8600) following the manufacturer's protocol. Briefly, 10 μl of MagneGST glutathione particles were washed three times with 100 μl of binding/wash buffer (4.2 mM Na_2_HPO_4_, 2 mM KHPO_4_, 140 mM NaCl, 10 mM KCl) to equilibrate them. Then, 100 μl of buffer (40 mM Tris-HCl pH 7.5, 80 mM NaCl, 8 mM MgCl_2_) containing 2.5 μg of protein (GST-Gα13iN or GST-PAK1-PBD (Cytoskeleton Inc., PAK-01-A)) along with 125 nM of mAAT-C_1-35_ or mAAT-C_1-17_ and mAAT-C_18-35_ peptides was mixed with the MagneGST glutathione particles. The mixture was incubated for 30 min at room temperature with gentle rotation to allow the binding of GST-tagged proteins and peptides to the beads. After incubation, the beads were washed three times with a binding/wash buffer to remove any non-specifically bound peptides. The bound peptides were then eluted by adding 100 μl of elution buffer (50 mM Tris-HCl, pH 8.1, 50 mM glutathione) and incubating the mixture at room temperature for 15 min with gentle rotation. The eluted peptides were desalted using SDB-XC StageTips (53) and dried using a vacuum centrifuge.

### Diazirine-based protein foot printing

Recombinant Human GNA13 protein (Tagged, 1 μg) (Abcam, ab268597) was dissolved in 220 μl of the buffer (40 mM Tris-HCl pH 7.5, 80 mM NaCl, 8 mM MgCl_2_, 10 mM 4-[3-(trifluoromethyl)-3*H*-diazirin-3-yl]benzoic acid and 10 mM NaOH) with or without 200 ng of mAAT_1-35_ peptide. 50 μl of the solution was placed in liquid nitrogen and irradiated by 365 nm ultraviolet light using a UV-LED irradiator (ULED-102CT, Shodensha) for 1 s to label the protein. After irradiation, the proteins were reduced by 10 mM dithiothreitol, alkylated by 50 mM iodoacetamide and digested by trypsin. The digests were desalted by StageTips and dried using a vacuum centrifuge. The samples were prepared in triplicate.

### LC/MS/MS analysis

The dried peptides from the affinity purification experiment were reconstituted in 50 μl of a solution containing 4% acetonitrile, 0.015% *n*-dodecyl-β-D-maltoside (54), and 0.5% trifluoroacetic acid. They were then analyzed by liquid chromatography/tandem mass spectrometry (LC/MS/MS) using a Q Exactive mass spectrometer coupled with an Ultimate 3000 RSLCnano LC pump (Thermo Fisher Scientific) and a PAL-xt HTC autosampler (CTC Analytics). The mobile phase A, consisting of 0.5% acetic acid, and the mobile phase B, consisting of 80% acetonitrile with 0.5% acetic acid, were used (55). The peptides were separated on a reversed-phase C18 column (100 μm × 15 cm) packed with Reprosil-Pur 120 C18-AQ 3 μm (Dr Maisch) using a 40-min linear gradient from 5% to 99% B at a flow rate of 500 nl/min. The mass spectrometer operated in data-dependent acquisition (DDA) mode, selecting the top 10 most intense precursor ions for fragmentation by higher-energy collisional dissociation (HCD) with a normalized collision energy of 27. The raw MS data were processed for peptide quantification based on peak areas of precursor ions using Skyline-daily (version 23.1.1.459) (56).

The dried peptides from the foot printing experiment were reconstituted in 7 μl of a solution containing 4% acetonitrile and 0.5% trifluoroacetic acid. They were analyzed by LC/MS/MS using an Orbitrap Fusion Lumos mass spectrometer coupled with the Ultimate 3000 RSLCnano LC pump and an HTC-PAL autosampler (CTC Analytics). The peptides were separated on a reversed-phase C18 column (100 μm × 15 cm) packed with Reprosil-Pur 120 C18-AQ 3 μm using a 20-min linear gradient from 5% to 40% B at a flow rate of 500 nl/min. The mass spectrometer operated in DDA mode, with a setting of 1.5 s cycle time. The precursor ions were fragmented by HCD with a normalized collision energy of 30. The peptides were identified by means of automated database search using Mascot (version 2.7.0) against the SwissProt Human database (accessed on 2019/10) with the enzyme specificity of trypsin with the allowance of up to 2 missed cleavages. The cysteine carbamidomethylation was set as the fixed modification. The methionine oxidation was set as the variable modification. The precursor ion peak areas of identified peptides and their diazirine modified forms (+202.024164) were quantified using Skyline-daily. Only one diazirine modification was allowed for a peptide. The raw MS data and analysis files have been deposited in the ProteomeXchange Consortium *via* the jPOST partner repository (https://jpostdb.org) (57) with the dataset identifier PXD054789.

### GTPase assays for G**α**13iN

The GAP and GEF assays for Gα13iN were performed using the GTPase-Glo assay (Promega, V7681) according to the manufacturer's instructions. The assay was performed in GAP or GEF buffer containing 2 μM untagged Gα13iN, 10 μM GST-p115-RH, 10 μM mAAT-C peptides, 10 μM GTP, 10 mM DTT at 25 °C for 120 min. Luminescence was measured by using multimode plate reader/ARVOX3 (PerkinElmer).

### GST pull-down assay

For the GST pull-down assay, GST-p115-RH protein (33 μg) was bound to 20 μl of glutathione-Sepharose 4B by gentle mixing in 200 μl of buffer A (20 mM HEPES, 5 mM βME, 1 mM MgCl_2_, 100 mM NaCl, 10 μM GDP, 0.1% C12E10, pH 8.0) for 60 min at 4 °C. The resulting GST-p115-RH beads were washed with buffer A, and incubated with untagged Gα13iN protein (2 μg) in buffer A with or without AlF_4_^-^ (30 μM AlCl_3_, 5 mM MgCl_2_, and 5 mM NaF) or mAAT-C peptides for 60 min at 4 °C. After three times washes with buffer A, the beads were resuspended in 20 μl buffer A and boiled in SDS sample buffer.

### Western blot

Equal amounts of protein samples were loaded on SDS-PAGE and transferred to Immobilon-P polyvinylidene difluoride (PVDF) membranes (Millipore). The PVDF membranes were blocked with Blocking-One (Nacalai Tesque) for 60 min. Primary antibodies were diluted with Blocking-One and incubated with the PVDF membrane for O/N at 4 °C. After three times washes with TBST, the membranes were incubated with horseradish peroxidase (HRP)-conjugated secondary antibodies (1:10,000, goat anti-mouse/rabbit NA931 V, NA934V; Cytiva) for 60 min at room temperature, washed three times with TBST, and treated with the chemiluminescence reagents (Western Lightning Plus-ECL (PerkinElmer) or WSE-7120LCP EzWestLumiplus (ATTO, 2332521)). Signals were detected with the LAS 4000 mini imaging system (Fujifilm) or the ChemiDoc Touch MP imaging system (Bio-Rad). Densitometry of immunoreactive bands was quantified using ImageJ software with the vehicle as an internal control. Primary antibodies included Anti-GNA13 antibody [EPR5436] (1:1,000, ab128900; Abcam) and Anti-RhoA antibody [EPR18134] (1:500, ab187027; Abcam). Uncropped Western blot images are shown in Figs. S2, S3, S4, S6 and S10 with the size markers.

### Cell culture

HeLa cells were cultured in Dulbecco's modified Eagle's medium (DMEM) supplemented with 10% fetal calf serum (FCS) (SIGMA, 19G00F) and 2 mM L-glutamine (Nacalai Tesque, 3953). Cell cultures were incubated at 37 °C in a humidified 5% CO_2_ atmosphere.

### RhoA activity assay

For the detection of intrinsic RhoA activity, HeLa cells were washed three times with phosphate-buffered saline (PBS) buffer and lysed in Pierce IP Lysis Buffer (Invitrogen, 87788) containing 1 mM DTT and 0.1 M PMSF. Cell lysates were centrifuged at 15,000 rpm for 15 min at 4 °C, and the supernatant was incubated with glutathione-Sepharose 4B and GST-Rhotekin-RBD protein (2 μg) (Cytoskeleton Inc, RT01) for 120 min at 4 °C. After three washes with Pierce IP Lysis Buffer, the beads were resuspended in 20 μl Pierce IP Lysis Buffer and boiled in SDS sample buffer. Samples were subjected to SDS-PAGE and immunoblotting using RhoA antibodies. Total cell lysates were used as loading controls (total RhoA).

### Plasma membrane recruitment assay for p115-RhoGEF

For the plasma membrane recruitment assay for p115-RhoGEF, HeLa cells (2000 cells/well) were seeded in a 96-well glass bottom culture plate (IWAKI 1860–096). After 24 h, cells were co-transfected with mTurquoise2Δ9-tagged Gα13 (Addgene, #112930) and SYFP1-p115-RhoGEF (Addgene, #112931) (50) by using PEI-MAX, according to the manufacturer's instructions (Invitrogen). After incubation for 24 h, the culture medium was replaced with serum-free medium, and the cells were treated with mAAT-C peptides for the indicated time. Cells were fixed with 4% paraformaldehyde in PBS buffer, permeabilized with 0.5% Triton X-100 in Tris-buffered saline for 15 min at room temperature and washed with PBS buffer. Cells were then blocked with Blocking-One for 60 min at room temperature and were counterstained with 4′,6-diamidino-2-phenylindole (1:1,000, D1306; Thermo Fisher Scientific). All images were acquired using an Olympus FV3000 confocal microscope with an x40 oil immersion objective. Random images were taken and the luminance of the plasma membrane and cytoplasm was analyzed using Olympus cellSense Count and Measure software. All experiments were repeated at least four times.

### Docking simulation of mAAT-C_1-17_ with G**α**13iN

The initial structure for the MD simulation of Gα13iN・GDP-mAAT-C_1-17_ complex was prepared by docking simulation using the program, HADDOCK2.4 (47). The input structure of Gα13iN・GDP was derived from the crystal structure in complex with p115RhoGEF RGS domain (PDB entry: 3AB3). The conformational space of mAAT-C_1-17_ was explored by T-REMD simulation starting from the extended structure and the two most stable structures were employed as the input structures. The extended structure was prepared by the program CNS 1.3 (58) In the T-REMD simulation, 102 replicas were used with temperatures ranging from 298 K to 500 K, and the replica exchange was attempted every 2 ps. A set of temperatures to obtain an exchange probability of 0.2 was generated using the Temperature generator for REMD simulations (59). 30 ns simulations were performed for each replica, and the total simulation time was 3.06 μs (30 ns × 102 replicas). In the docking simulation, active residues, which are presumed to be involved in the binding interface, were selected based on the diazirine-based protein foot printing data. All residues of mAAT-C_1-17_ was set as fully flexible segments. The resulting 400 models were ranked based on the HADDOCK score and the best model was adopted as an initial structure for the subsequent MD simulation. In addition to the score, the predicted binding mode of the representative models is evaluated by 50 ns MD simulations, of which conditions are identical to those described in the following “MD simulations” section. The representative models are each best model of top six clusters calculated by RMSD-based clustering with 5 Å cutoff.

### MD simulations

All MD simulations were carried out with periodic boundary conditions (PBC) by GROMACS 2019.1 (60) on an NVIDIA GeForce GTX 1080 GPU. Hydrogen atoms were added to the docking model and the topology files were generated by using a pdb2gmx module in the GROMACS 2019.1. The electrostatic potential for GDP was calculated at the RHF/6–31G∗ level using the General Atomic and Molecular Electronic Structure System (GAMESS) program (61), after which the atomic partial charges were assigned by the restrained electrostatic potential (RESP) approach (62). Other parameters for GDP were determined by the general Amber force field (GAFF) using the antechamber module of AMBER Tools (63, 64). The Amber ff99SB-ILDN force field was used for proteins and ions (65), and TIP3P potential was used for water molecules (66). Electrostatic interactions were calculated using the particle mesh Ewald (PME) method (67) with a cutoff radius of 10 Å, and a nonbonded cut-off of 10 Å was applied for van der Waals interactions. The P-LINCS algorithm was used to constrain all bond lengths to their equilibrium values (68). After energy minimization of the fully solvated models, the resulting systems were equilibrated for 100 ps under constant number of molecules, volume and temperature (NVT) conditions, followed by a 100 ps run under constant number of molecules, pressure and temperature (NPT) conditions, with the heavy atoms of the molecules held in fixed positions. The temperature was maintained at 298 K by velocity rescaling with a stochastic term (69), and Parrinello–Rahman pressure coupling (70) was used to maintain the pressure at 1 bar, with the temperature and pressure time constants set to 0.1 ps and 2 ps, respectively. Subsequently, 500 ns production runs with five different initial velocities were performed under NPT conditions.

## Data availability

The raw MS data and analysis files have been deposited in the ProteomeXchange Consortium *via* the jPOST partner repository (https://jpostdb.org) with the dataset identifier PXD054789.

All other data are contained in the manuscript.

## Supporting information

This article contains supporting information (Supplementary Figs. S1–S11).

## Conflict of interests

The authors declare that they have no conflicts of interest with the contents of this article.
