# Supplementary material for: Receptor-independent regulation of Gα13 by alpha-1-antitrypsin C-terminal peptides
Source: J Biol Chem. 2024 Dec 25;301(2):108136. doi: 10.1016/j.jbc.2024.108136 (PMC11815680; doi:10.1016/j.jbc.2024.108136)
Supplement: Supplemental Figure [file mmc1.pdf]

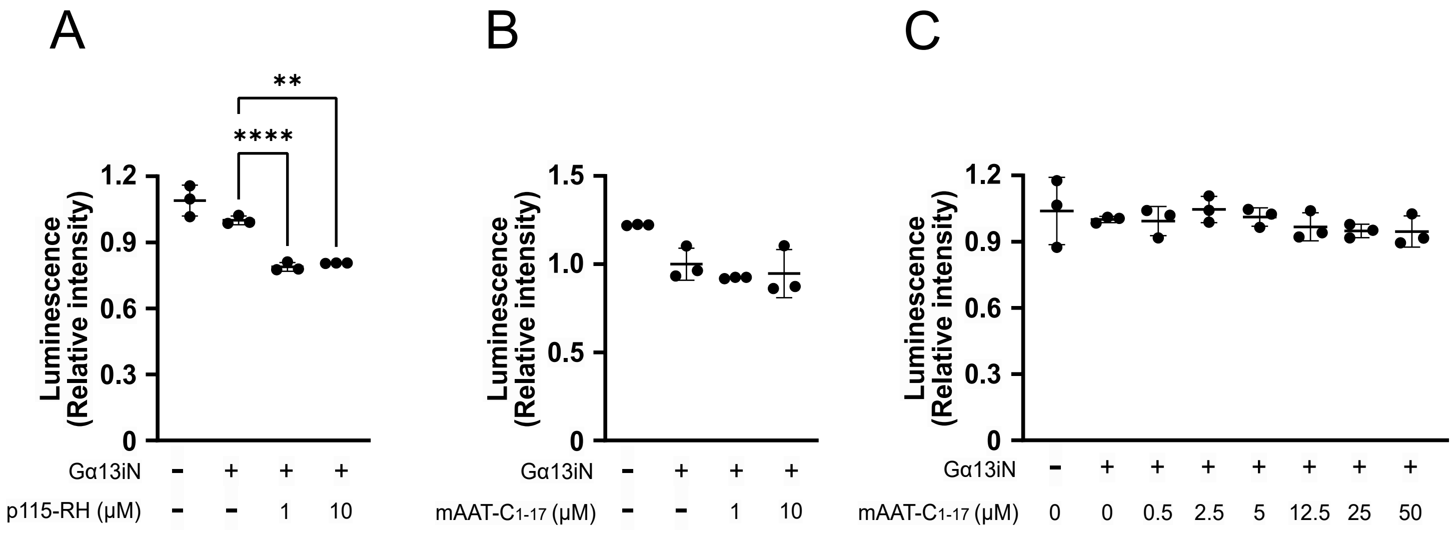

Supplementary Fig. 1 GTPase activity of Gα13

A, Effect of p115-RH on Gα13 GTPase activity. Untagged Gα13iN protein (2 μM) was incubated with or without p115-RH protein (1 μM or 10μM) in GAP buffer containing 10 μM GTP and 2 mM DTT (n=3 independent samples). **B,** Effect of mAAT-C_{1-17} on Gα13 GTPase activity in GAP buffer. Untagged Gα13iN protein (2 μM) was incubated with or without mAAT-C_{1-17} (1 μM or 10μM) in GAP buffer containing 10 μM GTP and 2 mM DTT (n=3 independent samples). **C,** Effect of mAAT-C_{1-17} on Gα13 GTPase activity in GEF buffer. Untagged Gα13iN protein (2 μM) was incubated with or without mAAT-C_{1-17} at the indicated concentrations in GEP buffer containing 10 μM GTP and 2 mM DTT (n=3 independent samples). Data were compared using Dunnett's multiple comparison test (mean ± S.D.) (A). ***p* < 0.01, *****p* < 0.0001.

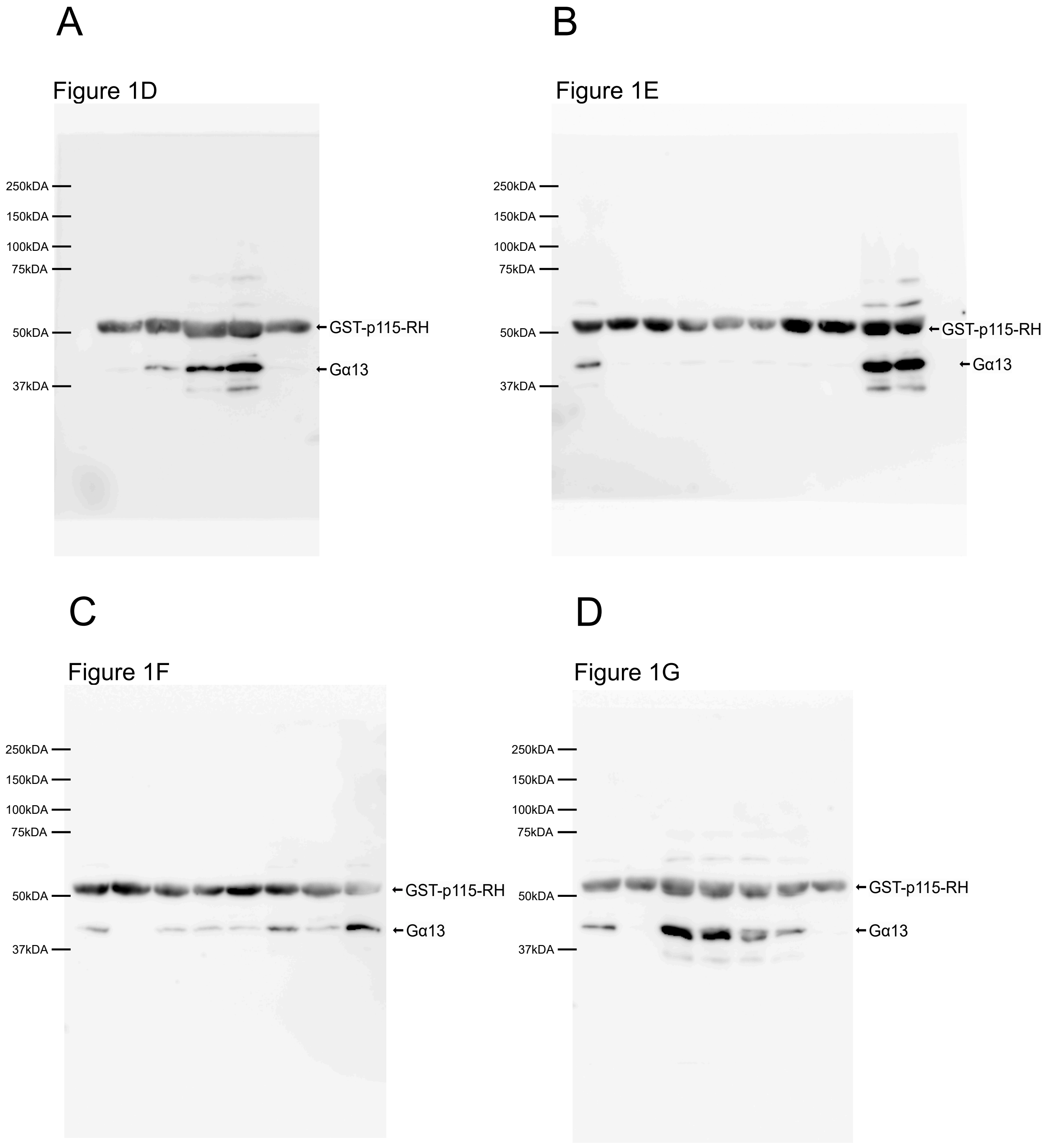

Supplementary Fig. 2

A-D, Uncropped Western blot images for Fig. 1D (**A**), Fig. 1E (**B**), Fig 1F (**C**), and Fig 1G (**D**).

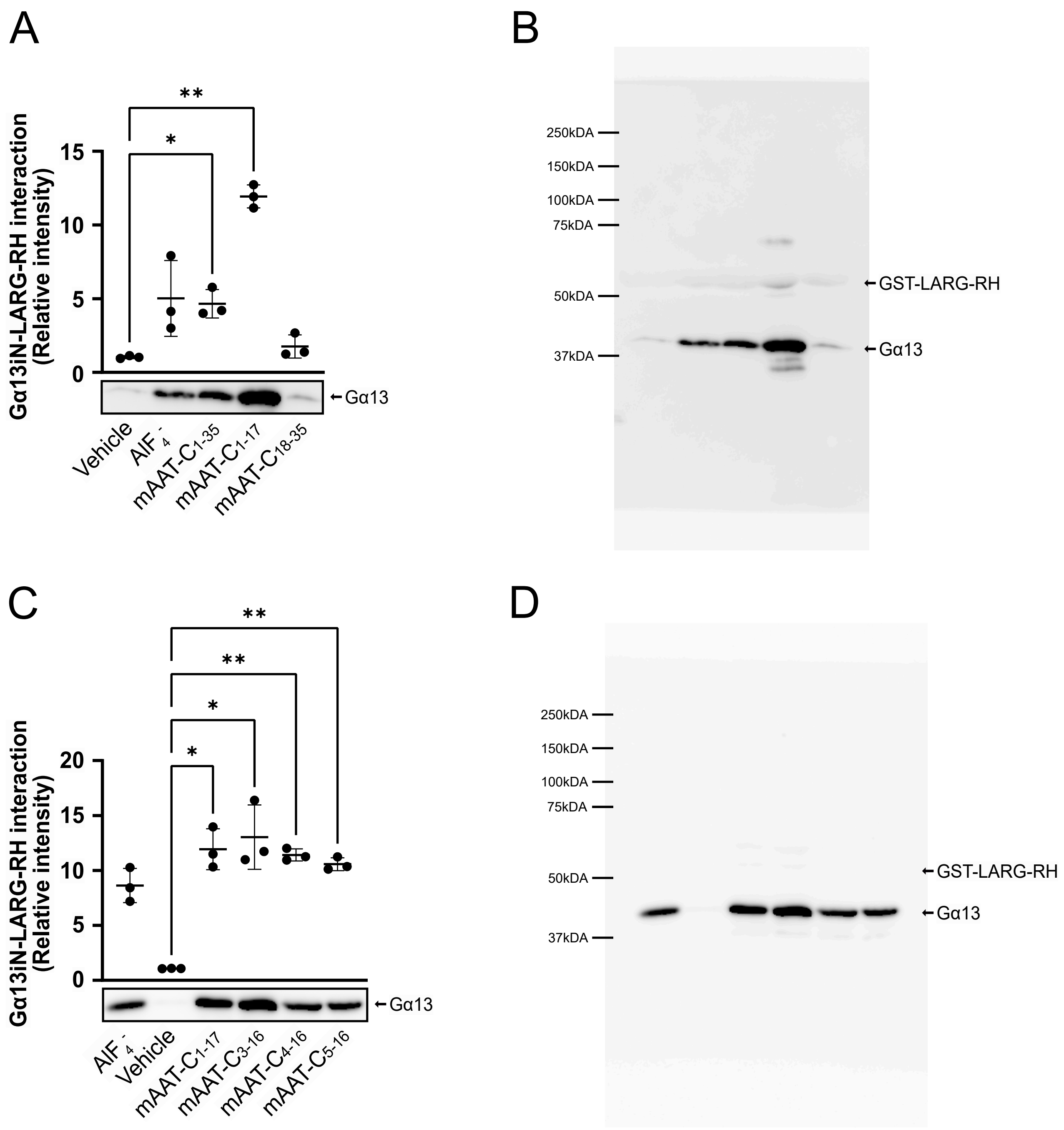

Supplementary Fig. 3 GST pull-down assay for Gα13･GDP to GST-LARG-RH

A, C, GST pull-down assay for Gα13･GDP to GST-LARG-RH with AlF_4^- or mAAT-C peptides. Precipitates were immunoblotted with G13 antibodies. Quantitative values for the Gα13iN bands are shown in the upper graph (n=3 independent samples). **B, D,** Uncropped Western blot images for (**A**) and (**C**). Data were compared using Dunnett's multiple comparison test (mean ± S.D.) (A, C). **p* < 0.05, ***p* < 0.01.

A

Figure 2A

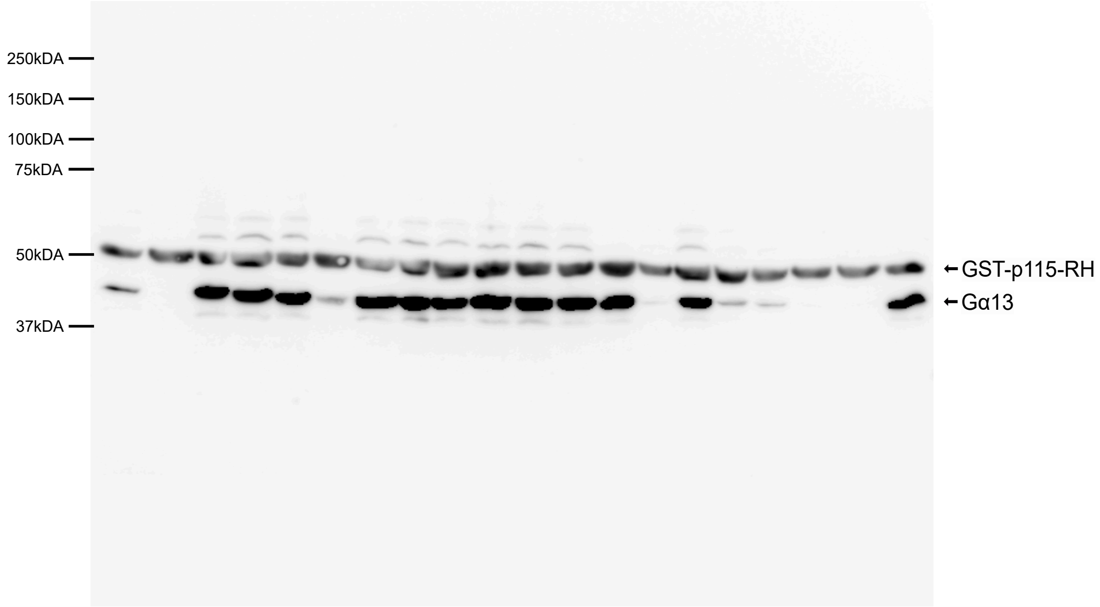

B

Figure 2C

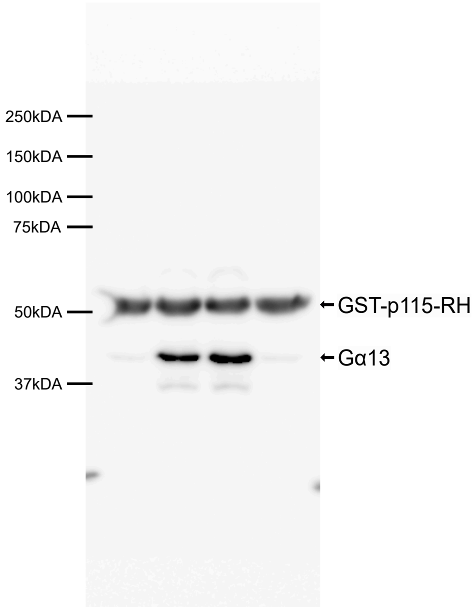

C

Figure 2D

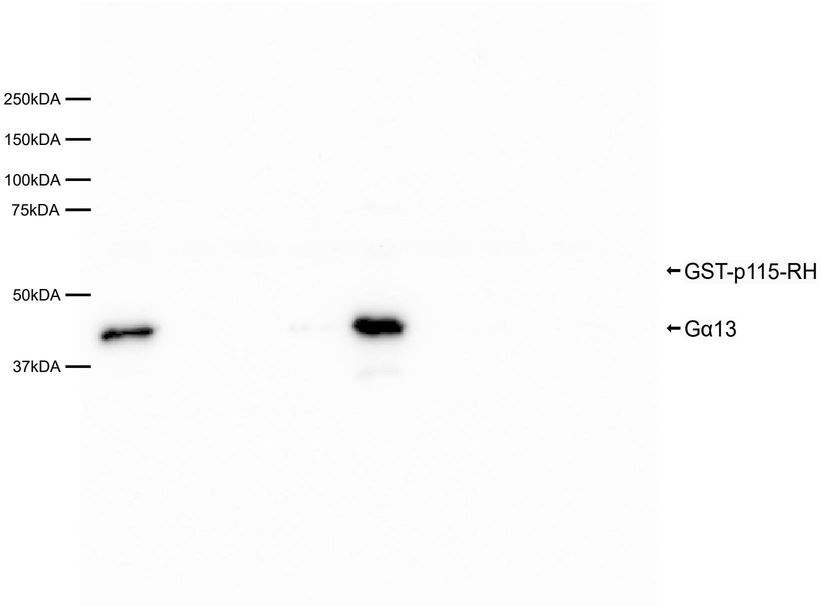

Supplementary Fig. 4

A-C, Uncropped Western blot images for Fig. 2A (**A**), Fig. 2C (**B**), and Fig 2D (**C**).

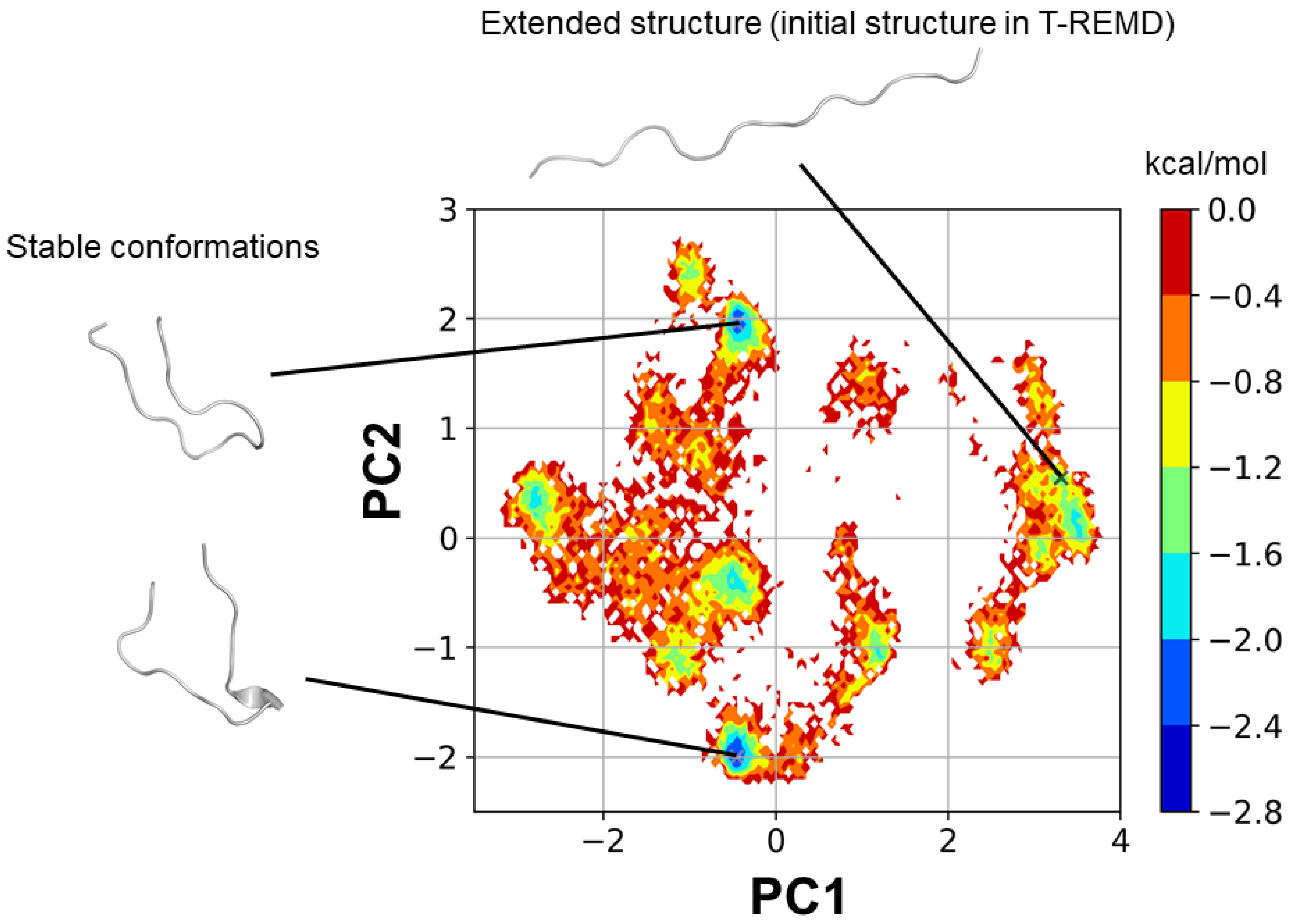

Supplementary Fig. 5 Conformational space of mAAT-C1-17 explored by T-REMD simulation starting from the extended structure.

The MD structures projected onto a principal component analysis (PCA) plane using the backbone dihedral angles as input variables. The free energy surface (in kcal/mol) was calculated from the conformational populations on the PCA plane, using the following equation: $\Delta G = RT\ln(P) - RT\ln(P_{min})$, where R is gas constant, T is temperature, P is the population at the indicated area, and $Pmin$ is the minimal population among them. The plot is gradually colored according to the free energy values as indicated by the color bar. The locations of the extended structure and the most stable conformations on the PCA plane are marked by ‘x’ symbols together with their tertiary structures.

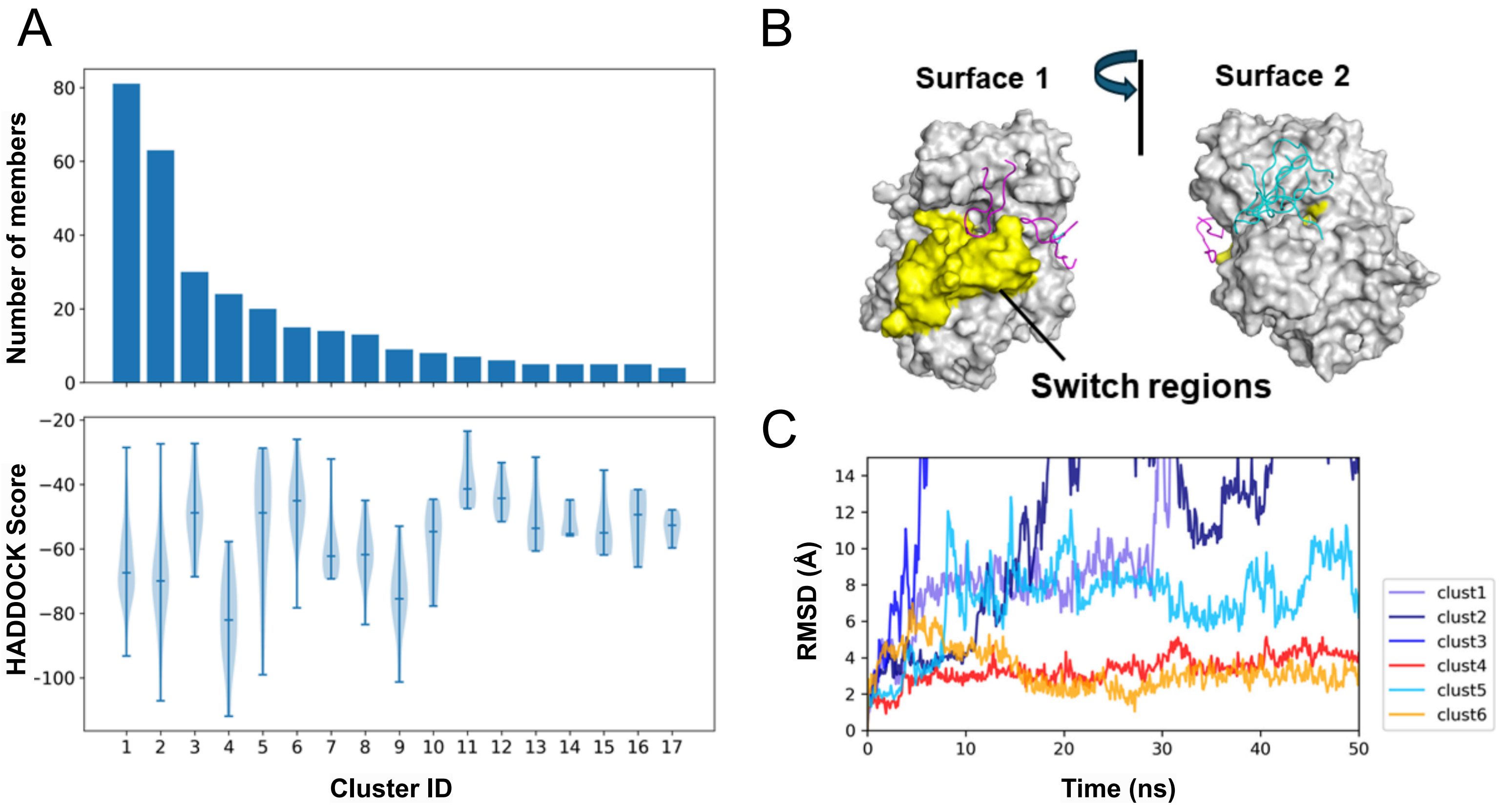

Supplementary Fig. 6 Analysis of docking models.

A, Statistic in each cluster derived from RMSD-based clustering. The number of members and distribution of HADDOCK scores in each cluster are indicated by a bar graph (upper panel) and a violin plot (lower panel), respectively. Lines in the violin plot indicate the minimum, maximum and median values. **B,** Representative binding modes derived from top six clusters. Gαi/13 and the bound peptides are represented by surface and ribbon models, respectively. mAAT-$C_{1\text{-}17}$ bound to Surface 1 (clusters 4 and 6) and Surface 2 (clusters 1, 2, 3, and 5) are colored by magenta and cyan, respectively. The three switch regions are highlighted in yellow. **C,** RMSD values for mAAT-$C_{1\text{-}17}$ in the representative docking models. The RMSD values are calculated for the Ca atoms of mAAT-$C_{1\text{-}17}$ from MD simulations with the length of 50 ns. The values calculated using the trajectories for each cluster model are indicated by a different color.

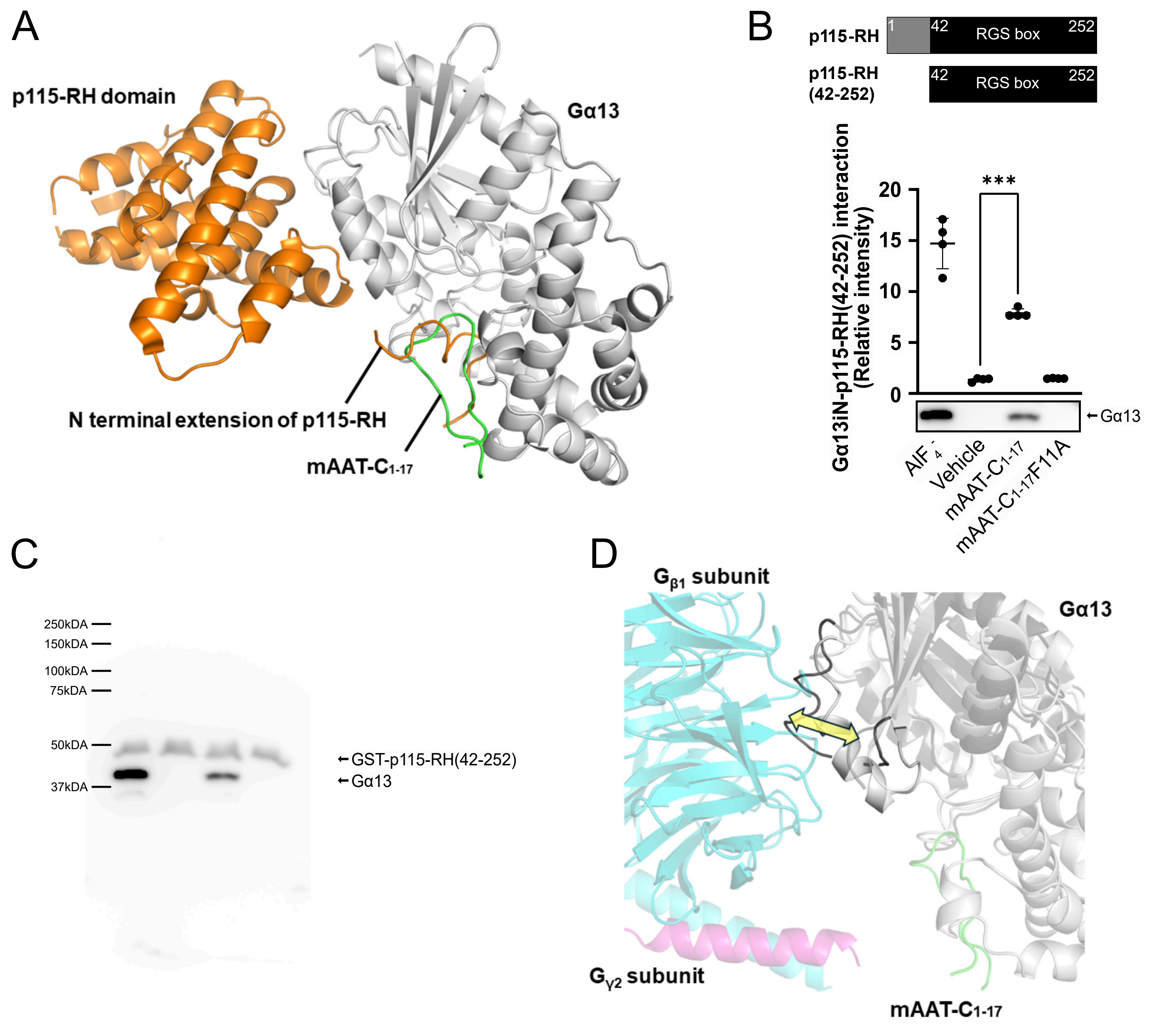

Supplementary Fig. 7 Comparison of the predicted binding site of mAAT-$C_{1\text{-}17}$ within Gα13 with those of the p115-RH and Gβ1/Gγ2 subunits.

A, Superimposition of the Gα13-mAAT-$C_{1\text{-}17}$ complex model with the crystal structure of the Gα13iN・GDP・AlF4-p115-RhoGEF-RH complex (PDB entry 3AB3). Gα13, mAAT-$C_{1\text{-}17}$, and p115-RH are colored grey, green, and orange, respectively. **B,** Schematic representation of the p115-RH and p115-RH(42-252) (top). GST pull-down assay for Gα13・GDP to GST-p115-RH(42-252) with AlF_4^- or mAAT-C peptides (bottom). The precipitates were immunoblotted with Gα13 antibodies. Quantitative values for the Gα13iN bands are shown in the upper graph (n=4 independent samples). **C,** Uncropped Western blot images for **(B)**. **D,** Superimposition of the Gα13-mAAT-$C_{1\text{-}17}$ complex model with Gα13-Gβ1/Gγ2 heterotrimer complexed with sphingosine 1-phosphate receptor 2 (PDB entry 7T6B). Gα13 complex with mAAT-$C_{1\text{-}17}$, mAAT-$C_{1\text{-}17}$, Gα13 in 7T6B, Gβ1, and Gγ2 are colored grey, green, black, cyan, and magenta, respectively. The conformational exchange in the switch II region is indicated by a yellow double-headed arrow. Data were compared using Dunnett's multiple comparison test (mean ± S.D.) (B). ***$p < 0.001$.

Supplementary Figure. 7

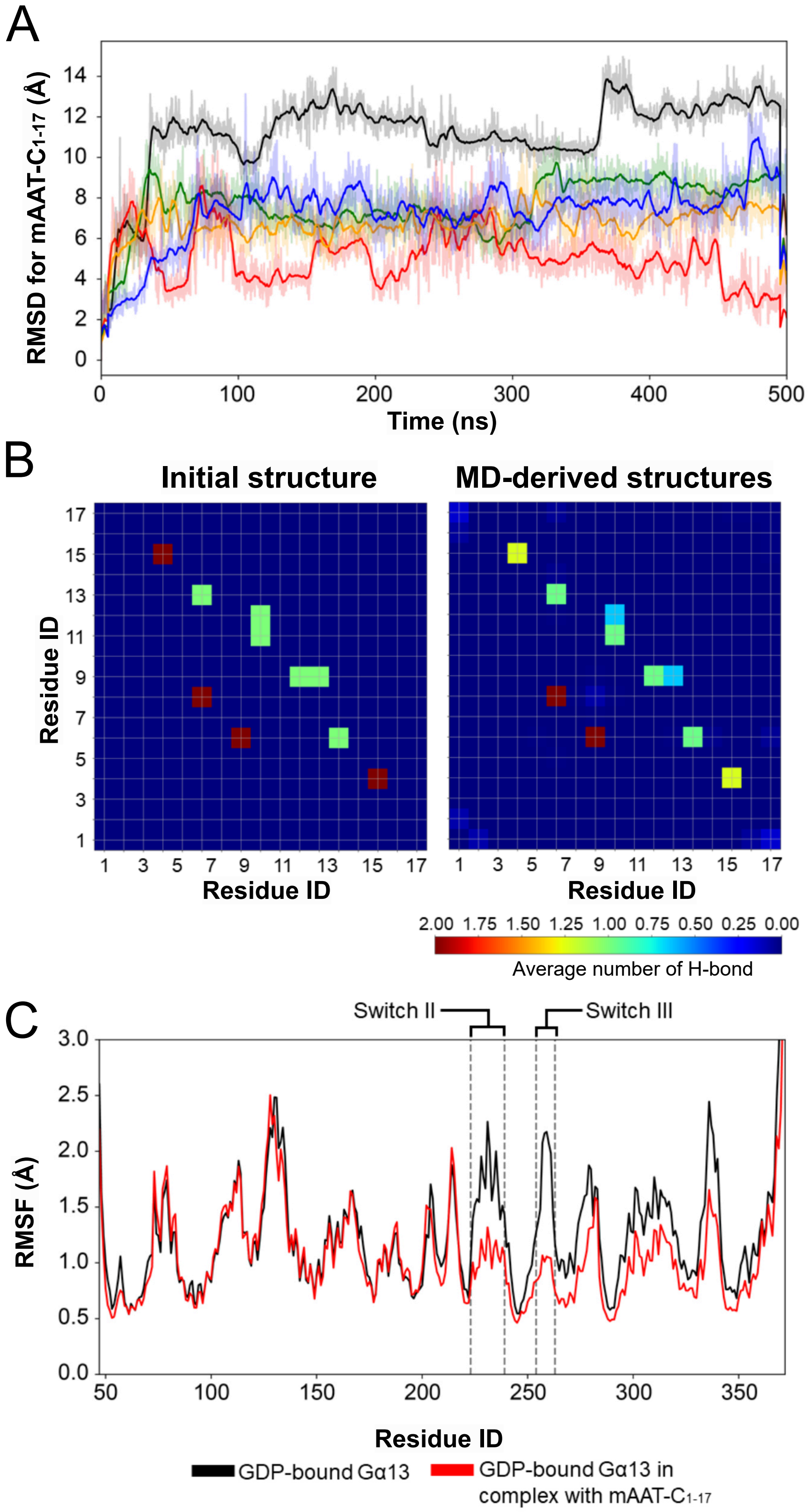

Supplementary Fig. 8 Dynamics behavior of Gα13 and mAAT-C1-17 in MD simulation.

A, RMSD values for mAAT-C1-17 in the complexed with Gα13. The RMSD values are calculated for the Ca atoms of mAAT-C1-17 from five independent simulations with the length of 500 ns. The five simulations were performed with different initial velocities and each is indicated by a different color. Five ns window averages are shown as thick lines. **B,** Hydrogen bonding interactions within mAAT-C1-17. The number of hydrogen bonding interactions is calculated for all residue pairs formed within mAAT-C1-17. Those found in the initial structure (left panel) and the averages across all MD trajectories (right panel) are visualized as heatmaps with a color gradient from blue (low) to red (high). The horizontal and vertical axes represent the number of residues. **C,** RMSF values for GDP-bound Gα13 in a free form (black) and in complex with mAAT-C1-17. The RMSF values for the free and complex forms are calculated for the Ca atoms as the average of the values derived from the five independent simulations and are indicated by the black and red lines, respectively. The switch region is indicated by the dashed grey line.

Supplementary Figure. 8

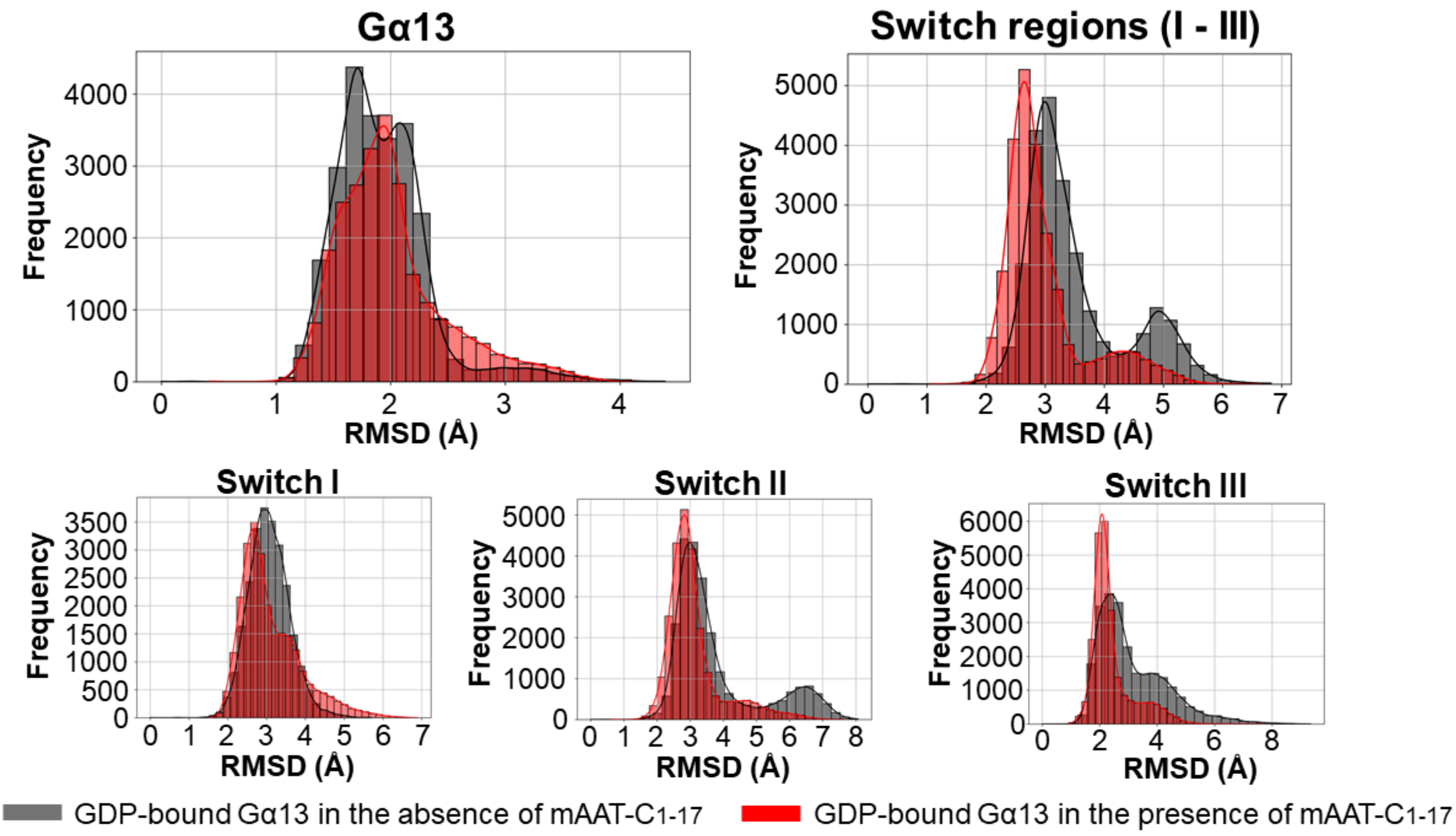

Supplementary Fig. 9 Comparison of the RMSD distributions of the total and switch regions of Gα13 in the presence or absence of mAAT-C1-17

RMSD distributions of the total (top left panel) and switch regions (top right and bottom panels) of Gα13 compared to the initial structure, i.e., the active conformation. The distributions in the presence and absence of mAAT-C1-17 are colored red and grey, respectively.

Supplementary Figure. 9

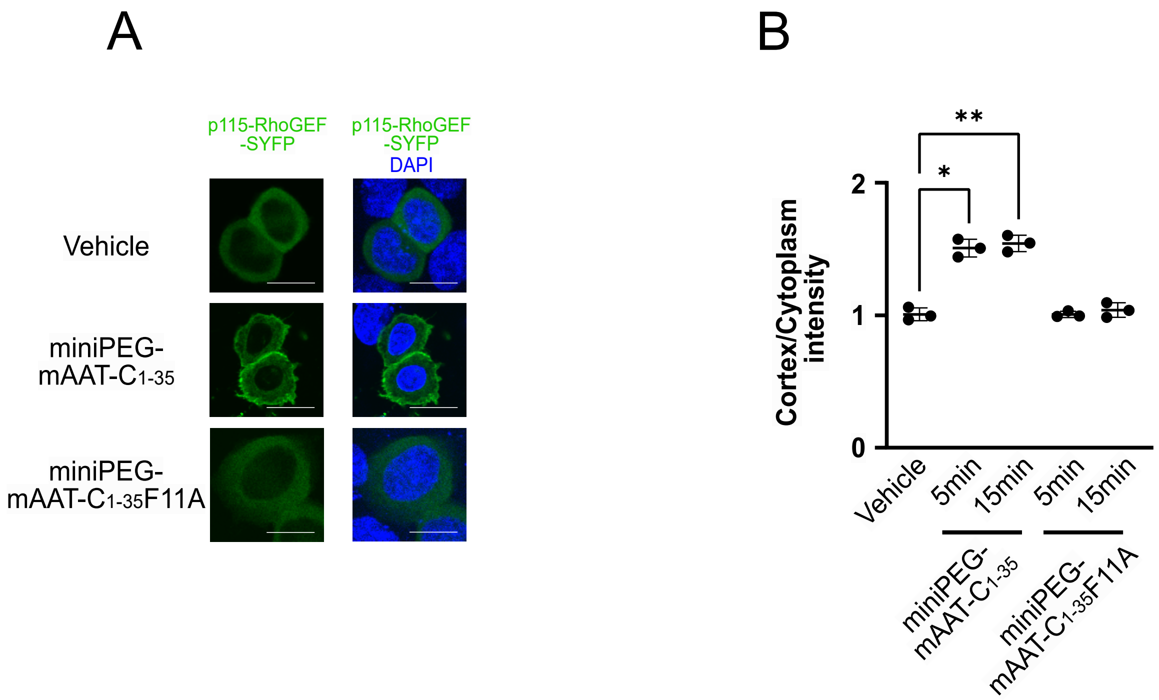

Supplementary Fig. 10 Plasma membrane recruitment assay of p115-RhoGEF

A, HeLa cells transfected with SYFP1-p115-RhoGEF were treated with or without mAAT-$C_{1\text{-}35}$ or mAAT-$C_{1\text{-}35}$F11A (50 μM) for the indicated time. Scale bars, 20 μm. **B,** The cortical intensity of SYFP1-p115-RhoGEF relative to the cytoplasmic intensity in (A) is shown. Each point represents the average of 11-14 cells/sample. (n=3 independent experiments). Data were compared using Dunnett's multiple comparison test (mean ± S.D.) (B). $^*p < 0.05$, $^{**}p < 0.01$.

A

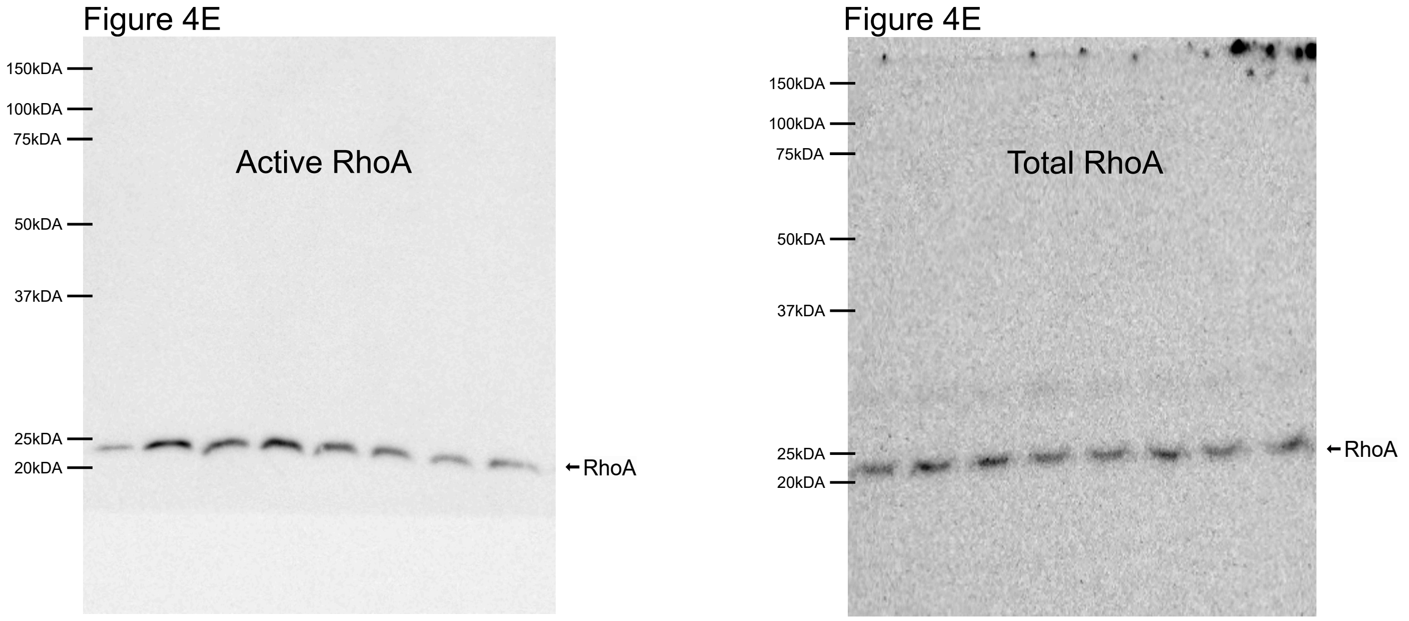

B

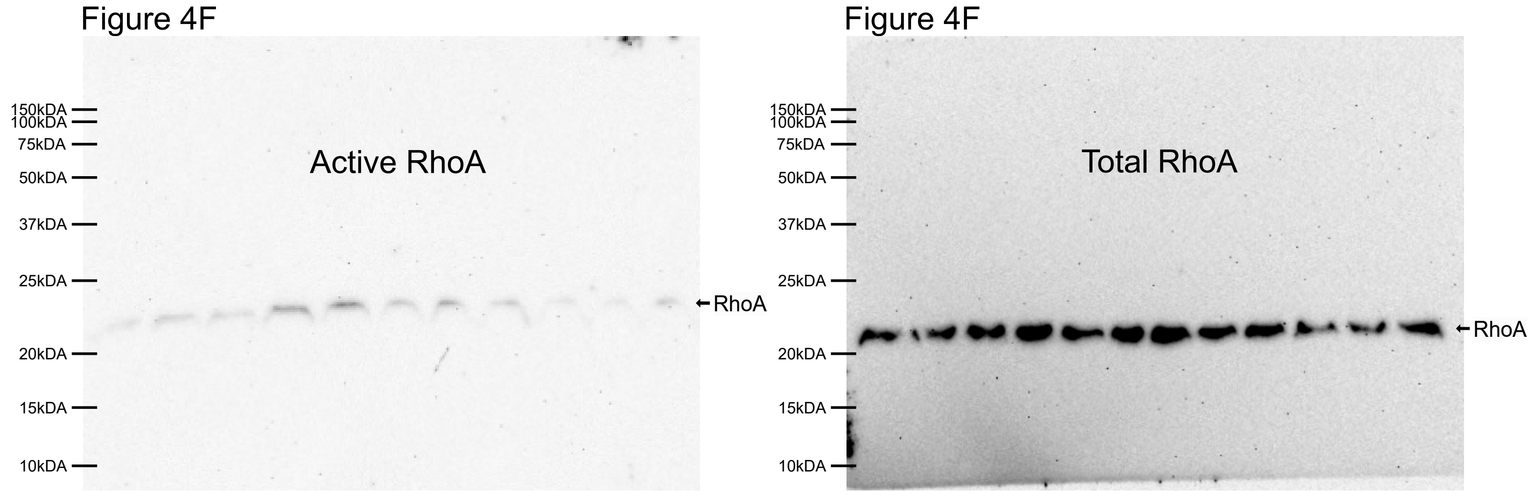

Supplementary Fig. 11

A, B, Uncropped Western blot images for Fig. 4E (**A**) and Fig. 4F (**B**).
